# Interaction of *Bacteroides fragilis* Toxin with Outer Membrane Vesicles Reveals New Mechanism of Its Secretion and Delivery

**DOI:** 10.3389/fcimb.2017.00002

**Published:** 2017-01-17

**Authors:** Natalya B. Zakharzhevskaya, Vladimir B. Tsvetkov, Anna A. Vanyushkina, Anna M. Varizhuk, Daria V. Rakitina, Victor V. Podgorsky, Innokentii E. Vishnyakov, Daria D. Kharlampieva, Valentin A. Manuvera, Fedor V. Lisitsyn, Elena A. Gushina, Vassili N. Lazarev, Vadim M. Govorun

**Affiliations:** ^1^Federal Research and Clinical Centre of Physical-Chemical Medicine Federal Medical Biological AgencyMoscow, Russia; ^2^Department of Polyelectrolytes and Surface-Active Polymers, Topchiev Institute of Petrochemical SynthesisMoscow, Russia; ^3^Department of Molecular Virology, FSBI Research Institute of Influenza, Ministry of Health of the Russian FederationSaint Petersburg, Russia; ^4^Lab of Genome Structural Organization, Institute of Cytology, Russian Academy of SciencesSaint Petersburg, Russia; ^5^Institute of Nanobiotechnologies, Peter the Great St. Petersburg Polytechnic UniversitySaint Petersburg, Russia; ^6^N.F. Gamalei Federal Research Centre for Epidemiology and Microbiology, Ministry of Health Russian FederationMoscow, Russia; ^7^Lab of Systems Biology, Moscow Institute of Physics and TechnologyDolgoprudny, Russia; ^8^Department of Proteomics, Shemyakin-Ovchinnikov Institute of Bioorganic Chemistry of the Russian Academy of SciencesMoscow, Russia

**Keywords:** toxin delivery, lipid protein interactions, shot gun lipidomics, Electron microscopy, fluorescence quenching, NMR

## Abstract

The only recognized virulence factor of enterotoxigenic *Bacteroides fragilis* (ETBF) that accompanies bloodstream infections is the zinc-dependent non-lethal metalloprotease *B. fragilis* toxin (BFT). The isolated toxin stimulates intestinal secretion, resulting in epithelial damage and necrosis. Numerous publications have focused on the interrelation of BFT with intestinal inflammation and colorectal neoplasia, but nothing is known about the mechanism of its secretion and delivery to host cells. However, recent studies of gram-negative bacteria have shown that outer membrane vesicles (OMVs) could be an essential mechanism for the spread of a large number of virulence factors. Here, we show for the first time that BFT is not a freely secreted protease but is associated with OMVs. Our findings indicate that only outer surface-exposed BFT causes epithelial cell contact disruption. According to our *in silico* models confirmed by Trp quenching assay and NMR, BFT has special interactions with outer membrane components such as phospholipids and is secreted during vesicle formation. Moreover, the strong cooperation of BFT with polysaccharides is similar to the behavior of lectins. Understanding the molecular mechanisms of BFT secretion provides new perspectives for investigating intestinal inflammation pathogenesis and its prevention.

## Introduction

Of the numerous microbial species that inhabit the gastrointestinal tract of mammals, Bacteroidetes is the most abundant gram-negative bacterial phylum (Ley et al., 2008). The anaerobe *Bacteroides fragilis* is a common colonic symbiont with an affinity for mucosal colonization, although it makes up only 1 to 2% of the cultured fecal flora (Huang et al., 2011). There are two molecular subtypes, non-toxigenic *B. fragilis* (NTBF) and enterotoxigenic *B. fragilis* (ETBF). ETBF is an intestinal bacterium that has been associated with inflammatory bowel disease and colorectal cancer in humans (Prindiville et al., 2000; Toprak et al., 2006). The only well-studied virulence factor specific to ETBF is the secreted metalloprotease *B. fragilis* toxin (BFT) (Moncrief et al., 1995; Franco et al., 1997). BFT can affect zonula adherens and tight junctions in the intestinal epithelium by cleaving E-cadherin (Wu et al., 1998), resulting in rearrangements of the actin cytoskeleton of epithelial cells. BFT is synthesized as a 44.4-kDa precursor (pBFT), which is then processed into a 21-kDa mature BFT (mBFT) that is secreted into the supernatant of cultured cells (Kling et al., 1997). Three toxin isoforms have been described, BFT1, BFT2, and BFT3, with isoform BFT2 being the most common (Scotto d'Abusco et al., 2000). Although BFT is a secreted protease, nothing is known about the mechanisms of its secretion and transport to host cells. Gram-negative bacteria have evolved mechanisms to deliver virulence factors to the host (Koster et al., 2000). Well-studied examples include type III secretion systems (Galán et al., 2014), type IV secretion systems (Wallden et al., 2010), and type VI secretion systems, which are required for virulence factor transport to host cells (Hachani et al., 2016). Genomic studies of *B. fragilis* have not shown evidence of type III, IV, autotransporter, or two-partner secretion systems (Wilson et al., 2015). However, *B. fragilis* was shown to possess genes for Hly type I secretion systems, which are similar to the hemolysin type I secretion system HlyDb of *Escherichia coli* (Wang et al., 1991). Type VI secretion systems (T6SS) were recently discovered in a few Bacteroidetes strains, thereby extending the presence of these systems beyond Proteobacteria. Comprehensive analysis of all sequenced human gut Bacteroidales strains has shown that more than half contain T6SS loci (Coyne et al., 2016). T6SS as a multiprotein complex is specially organized into three distinct genetic architectures (GA) where GA1 and GA2 loci are present on conserved integrative conjugative elements (ICE) and are transferred and shared among diverse human gut Bacteroidales species. But GA3 loci are not contained on conserved ICE and are confined to *B. fragilis*. Thorough research has showed that GA3 loci of *B. fragilis* could be a source of numerous novel effector and immunity proteins (Chatzidaki-Livanis et al., 2016). But there is no evidence that T6SS may be used for *B. fragilis* toxin secretion.

Rather than secrete virulence factors into the surrounding milieu, where they could be degraded by host proteases, many gram-negative pathogens utilize outer membrane vesicles (OMVs) as a mechanism of delivering active proteins and other moieties into host cells (Kulp and Kuehn, 2010). Toxin delivery mediated by OMVs is recognized as a potent virulence mechanism for many pathogens (Ellis and Kuehn, 2010). It is now well known that both non-pathogenic and pathogenic gram-negative bacteria constitutively release OMVs (Kuehn and Kesty, 2005). OMVs are spherical proteoliposomes that have an average diameter ranging from 20 to 150 nm and that are enriched with outer membrane proteins, phospholipids, polysaccharides, and numerous proteins of a wide molecular mass range (Mashburn-Warren et al., 2008). Many periplasm-located virulence factors are enriched in OMVs, including Shiga toxin produced by *E. coli* and Cag toxin produced by *Helicobacter pylori* (Ismail et al., 2003; Kesty and Kuehn, 2004). The large number of enzyme-containing OMVs produced by *B. fragilis* suggests that OMV transport may be an important export pathway (Patrick et al., 1996; Cerdeño-Tárraga et al., 2005). Intracellular, periplasmic and outer membrane-bound proteases have been identified in *B. fragilis* (Elhenawy et al., 2014). Moreover, *B. fragilis* OMVs which contain surface located polysaccharide A have been shown to play an anti-inflammatory role by acting on regulatory T (Treg) cells (Shen et al., 2012). Many hydrolytic enzymes, which are generally considered pathogenic factors, may be bound to the membrane and/or secreted (Gibson and Macfarlane, 1988). Toxins secreted by enterotoxigenic *E. coli* and *V. cholerae* have been found to associate with LPS on the outer surface of OMVs (Horstman and Kuehn, 2002; Chatterjee and Chaudhuri, 2011), in a manner similar to lectins. Lectins are usually characterized by weak interactions between carbohydrates and proteins through hydrogen and coordinate bonds (Richards et al., 1979). Because the *B. fragilis* genome does not encode for known secretion system genes involved in BFT secretion and transport, we reasoned that BFT might be delivered to host cells by OMVs. Consequently, the toxin is incorporated in vesicle membranes during membrane formation due to interactions with outer membrane components, such as lipids and LPS. In the present study, we demonstrate that BFT is associated with OMV membranes through hydrophobic and electrostatic interactions and forms hydrogen and coordinate bonds with membrane components, similar to the behavior of lectins.

## Results

### OMV production by NTBF and ETBF and BFT2 detection in association with OMVs

**Figure 1:**
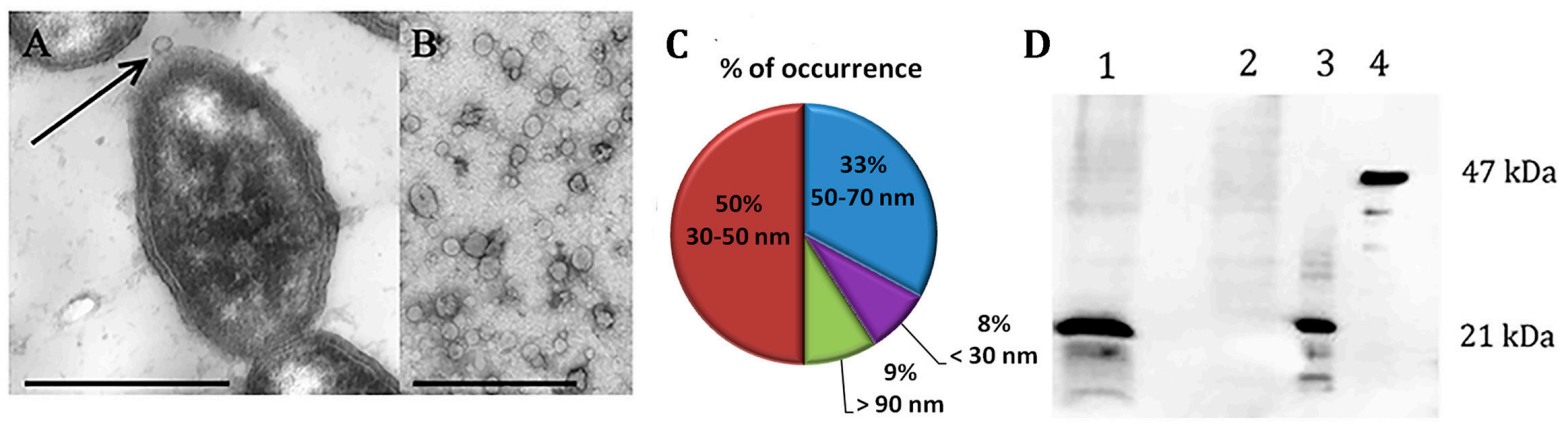
***B. fragilis* produces outer membrane vesicles (OMV), containing BFT**. TEM of thin section of ETBF cells during vesicle production **(A)** and isolated ETBF OMVs **(B)**, at magnification: x 10,000. Scale bars represent 1 μm for **(A)** and 300 nm for **(B)**. **(C)** OMV size distribution diagram determined from measurements of about 1000 OMVs from 10 samples **(D)**–5 μg of extracted OMV proteins of each strain (ETBF–1; NTBF–2) were run on 10% SDS-PAGE followed by western blot with antibody against BFT2. 100 ng of each recombinant forms of the toxin were also added (3–mBFT2 and 4–pBFT2).

To analyze the surface structure of both NTBF and ETBF strains, we examined the bacteria by TEM, which revealed the formation of OMVs on the cell surface (Figure 1A). It was observed that both strains (ETBF and NTBF) of *B. fragilis* produce vesicles (Figures 1A,B for ETBF and Supplementary Figures 2A,B for NTBF) with sizes ranging from 20 to 100 nm (Figure 1C). To avoid contamination with bacteria, small volume of OMV containing solution were cultivated on blood agar plates under anaerobic conditions for 2 days. *B. fragilis* colonies were not observed on blood agar plates.

After isolation of OMV containing fraction from 250 ml cultured cells, we measured the total protein concentration of the ETBF and NTBF OMV preparations, which was ~40–60 μg in 50 μl. For toxin detection, we analyzed 40 μg ETBF and NTBF OMV preparations by western blot using polyclonal antibodies (anti-BFT2) specific to mBFT2 and pBFT2. As a result, we observed the association of mBFT2 with ETBF vesicles (Figure 1D). OMV-depleted medium was also used for toxin detection. Briefly, 100 ml OMV-free medium was lyophilized under reduced pressure by a rotary evaporator and analyzed by western blot with polyclonal antibodies (anti-BFT2). We did not detect any visible amount of free toxin. pBFT2 was not detected in the OMV fraction or in OMV-depleted medium. The cells fractions were also examined for toxin existence by western blot using polyclonal antibodies (anti-BFT2) specific to mBFT2 and pBFT2. As a result we detected major part of the pBFT2 in membrane fraction and small amount of pBFT2 in periplasm fraction of ETBF cells (Supplementary Figures 1A,B). We examined cells fractions by Q-TOF analysis (Supplementary Information 1).

**Figure 2:**
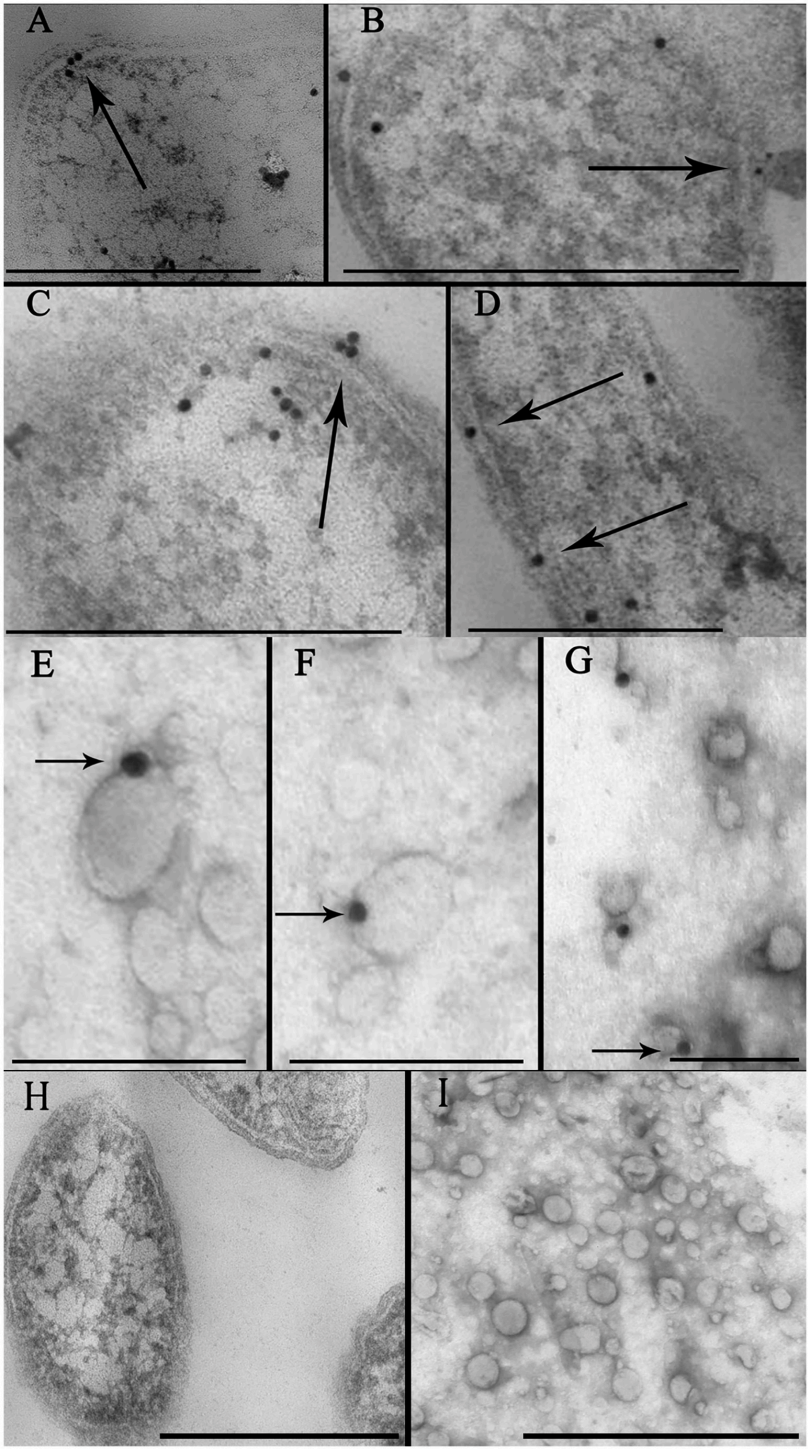
**Identification of toxin cell and OMV localization via immune-microscopy with antibodies against BFT**. **(A–D)** Thin sections of ETBF cells analyzed by TEM contain some labels (black dots) coupled with antibodies against BFT fixed on periplasm and membrane (indicated by arrow). Several labels coupled with antibodies against BFT fixed on cytoplasm indicating probable toxin formation. Scale bars represent 300 nm for **(A)** and **(D)**, 500 nm for **(B)** and **(C)**. **(E–G)**—Negatively stained OMV preparations isolated from ETBF culture and coincubated with labels, (black dots indicated by arrow) coupled with antibodies against BFT were analyzed by TEM. Scale bars represent 100 nm for **(E,F)** and 150 nm for **(G). (H)** Thin section of NTBF cells analyzed by TEM contains no fixed labels coupled with antibodies against BFT. **(I)** Negatively stained OMV preparations isolated from NTBF culture contain no fixed labels coupled with antibodies against BFT. Scale bars represent 600 nm for **(H)** and 400 nm for **(I)**.

To confirm the localization of the toxin in the bacterial membrane and in the OMV preparation, we performed immunoelectron microscopic analyses of thin sections using gold nanoparticles coupled with anti-BFT2 antibodies. We detected gold particles coupled with anti-BFT2 antibodies on the membrane and in the periplasm of ETBF (Figures 2A,C, **indicated by arrow**), which confirmed the previous results obtained by western blot analysis. There was no labeling of the same structure in NTBF cells (Figure 2H and Supplementary Figure 2A). We observed several labels located in the cytoplasm of bacterial cells, potentially indicating toxin formation (Figures 2B–D). Dynamics of OMV formation and release from the bacterial cell surface were monitored (Figure 2B, **indicated by arrow**). Importantly, we observed label predominantly located on the outer membrane (Figure 2D, **indicated by arrow**). ETBF and NTBF OMV samples were negatively stained and labeled with anti-BFT2 antibodies coupled with gold particles. We also examined freely isolated OMVs preparation for toxin localization by immunogold staining. We observed that the label was located on the surface of the ETBF OMV, indicating that the toxin was associated with the outer membrane (Figures 2E–G). There was no labeling of the same structure in NTBF OMVs (Figure 2I and Supplementary Figure 2B).

### Shot-gun lipidomics of the *B. fragilis* outer membrane

**Figure 3:**
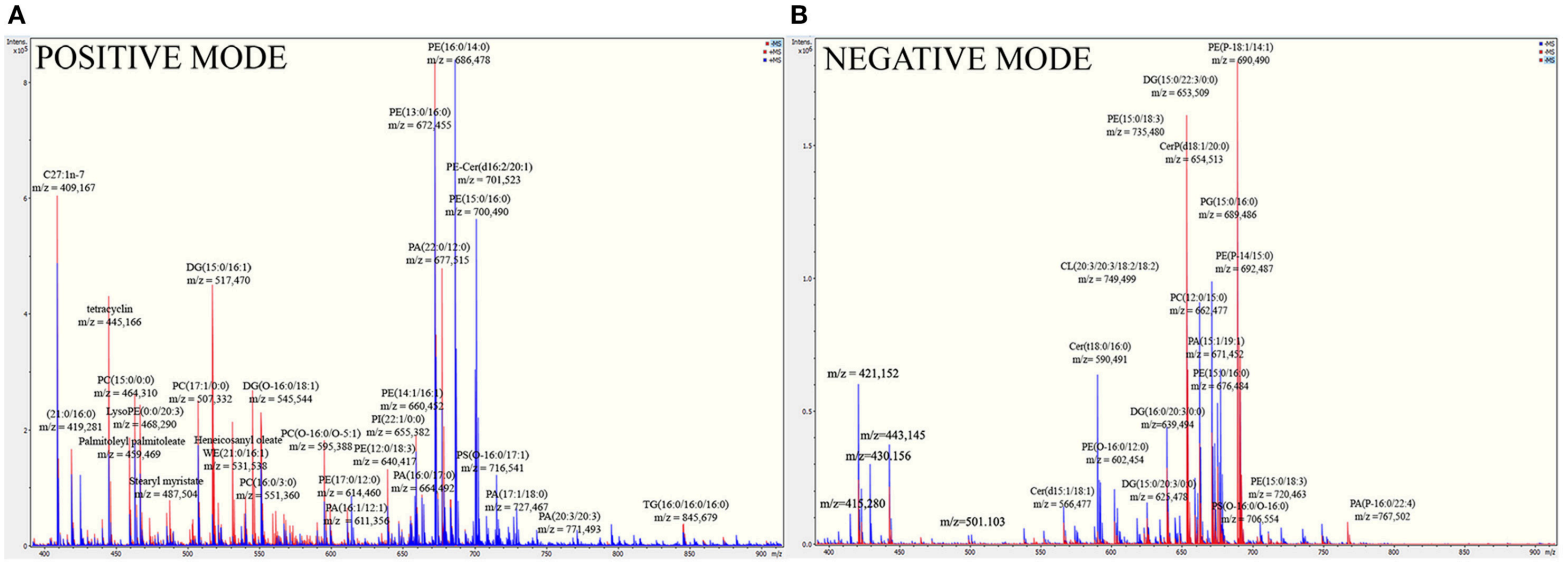
**Full mass spectra of *B. fragilis* lipids show the most represented lipids of *B. fragilis* membrane**. **(A)** MS analysis of ETBF (colored in red) and NTBF (colored in blue) membrane in positive linear mode. **(B)** MS analysis of ETBF (colored in red) and NTBF (colored in blue) membrane in negative linear mode. *B. fragilis* cells membrane lipids were extracted by Folch method (see material and methods), dissolved in 200 μl ethanol and analyzed by quadruple time-of-flight tandem mass spectrometer. Mass spectrum of ETBF and NTBF in positive and negative linear mode showed the same cluster of peaks, indicating the most represented classes of lipids (PC, PE, DG, PA).

To detect the components of the *B. fragilis* membrane, which the toxin could potentially interact with, shot-gun mass spectrometric analysis of the isolated membrane lipids and LPS extracts was performed. In the fragmentation spectra of membrane lipids, we primarily detected characteristic ions of polar groups of phospholipids, variable fatty acid residues capable of forming different diacylglycerol esters, cycles, and other compounds with water loss. The full list of the identified ions is shown in Supplementary information 2 (Table 1). As expected, the most represented ions in the spectrum belonged to the classes of phosphatidylethanolamine (PE), phosphatidylcholine (PC), and diacylglycerol (DG) (Figure 3).

To determine the specific molecular structure of *B. fragilis* LPS, we performed comprehensive mass spectrometry analysis. We used the Tri-Reagent method to extract LPS from *B. fragilis* cell membranes (Elhenawy et al., 2014). To profile the extracted LPS, we used direct injection electrospray-time-of-flight mass spectrometry in negative ion mode as described in the **Materials and Methods** section. As a result, we identified 38 different LPS compounds in the *B. fragilis* LPS preparation [Supplementary information 2 (Table 2)]. Among the detected ions, we reliably identified lipid A-disaccharide-1-P, 3-deoxy-D-manno-octulosonyl-lipid IV(A), KDNalpha2-3Galbeta1-4Glcbeta-Cer[d18:1/24:1(15Z)] and numerous galactosylceramides and glucosylceramides (polysaccharide chains consisting of 4 to 8 carbohydrate units). The identified lipid A-disaccharide-1-P structure was the same as that previously described by Elhenawy et al. (Patrick et al., 1996). As expected, the most represented ions in the spectrum belonged to classes of cerebrosides that are normally produced by *B. fragilis* cells. The mass spectrometry metabolomic data have been deposited to UCSD Center for Computational Mass Spectrometry (MassIVE ID: MSV000080382).

### Modeling and docking of phospholipids (PC and PE), lipid A, polysaccharide chain, and BFT2 (mBFT2, pBFT2)

Two phospholipids of the most abundant classes represented in *B. fragilis* membranes (PC and PE) according to our shot-gun lipidomics data and the main components of LPS [lipid A and polysaccharide chain (PSC)] were docked to pBFT2 and mBFT2 surfaces. Modeling of the pBFT2 and mBFT2 surfaces according to hydrophobic potential distribution, cavities, and potential donors and acceptors of hydrogen bond distribution is shown in Supplementary Figures 3, 4.

**Figure 4:**
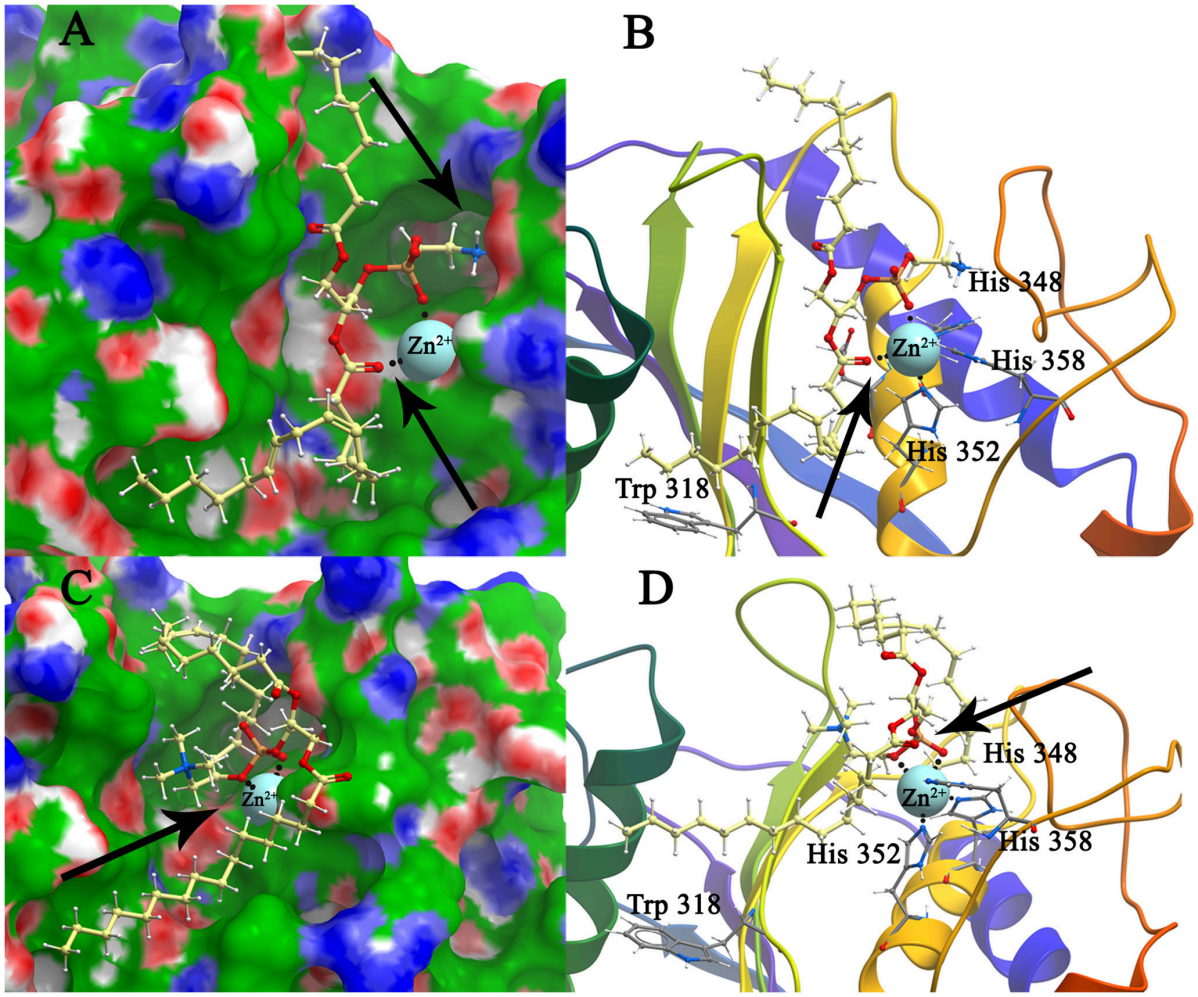
**Structure model of mBFT and best (according to the binding energy) ligands (A)**—PC and **(C)**—PE location on the mBFT Conolly surface, painted depending on the values of the hydrophobic potential—green, donors—blue, acceptors—red, resulting docking via ICM. The atoms of the ligands are painted in the following colors: polar hydrogens—blue, nitrogen—dark blue, oxygen—red, phosphorus—orange, carbon—white. Zn ions are painted in blue. The non-polar hydrogens not shown. Black dot lines indicate hydrogen bonds. Oxygen of lipid phosphate group colored in red forming coordination bonds with zinc ion. Nitrogen group of PE forming donor-acceptor bounds with amino acids indicated by arrow. Detailed image of mBFT active center with PC **(B)** and PE **(D)**.

**Figure 5:**
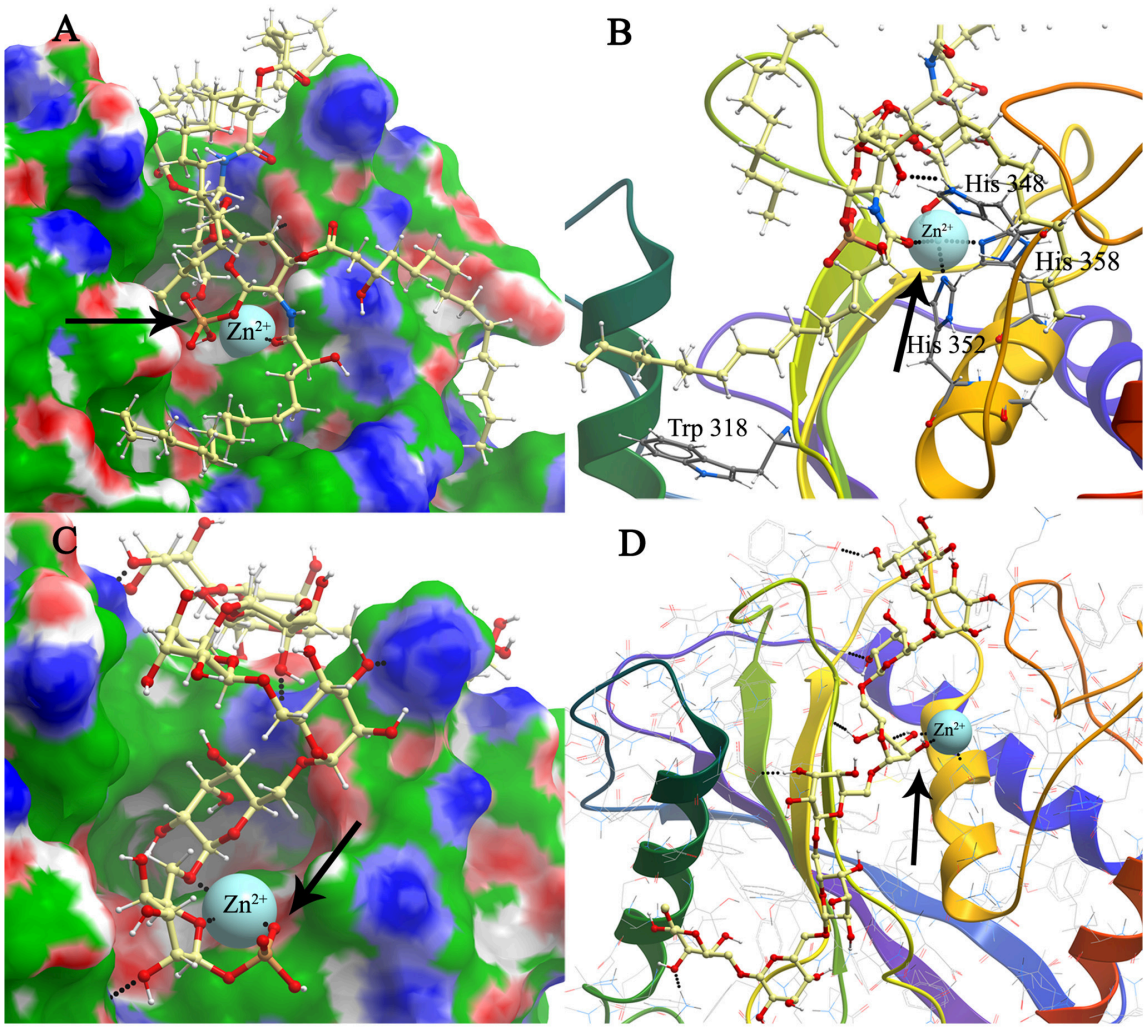
**Modeling and docking of protein-lipid A and polysaccharide chain association**. Structure model of mBFT and best (according to the binding energy) ligand–lipid A **(A)** and polysaccharide chain **(C)** location on the mBFT Conolly surface, painted depending on the values of the hydrophobic potential—green, donors–blue, acceptors–red, resulting docking via ICM. Oxygen of lipid phosphate group colored in red forming coordination bonds with zinc ion (indicated by arrow). The atoms of the ligands are painted in the following colors: Polar hydrogens–blue, nitrogen–dark blue, oxygen–red, phosphorus–orange, carbon–white. Zn ions are painted in blue. The non-polar hydrogens not shown. Black dot lines indicate hydrogen bonds. Oxygen of lipid phosphate group colored in red forming coordination bonds with zinc ion. Detailed image of mBFT active center with lipid A **(B)** and polysaccharide chain **(D)**.

The conformations of mBFT2 complexes with PE, PC, lipid A, and PSC with best values of binding energies are shown in Figures 4, 5. As is evident from **S**upplementary information 2 (Table 3), hydrophobic interactions as well as electrostatic make a significant contribution (Δ***G***_*non*−*polar*_) to the binding energy upon complex formation for both proteins (mBFT2 and pBFT2). The Coulomb contribution (Δ***G***_*el*_) in the case of pBFT2 complex formation is insignificant (Δ***G***_*el*_ comparable to Δ***G***_*non*−*polar*_) compared with mBFT2 complex formation due to the latter's interaction with the zinc ion. Furthermore, the loss of conformational mobility during binding by the ligand as well as by the target leads to a decrease in entropy (TΔS) which reduces binding energy. Also note that electrostatic component of solvation energy (Δ*G*_*polar*_) significantly decreases binding energy.

Lipids can form H-bonds with amino acid residues on pBFT2/mBFT2 groove surfaces (Supplementary Figures 5, 6, PC acts as an acceptor, and PE can be either a donor or an acceptor).

Importantly, the nitrogen group of PE tends to form donor-acceptor bonds with amino acids located in the mBFT2 cavity (Figure 4A, **indicated by arrow**). In contrast, hydrophobic contributions are greater in the case of pBFT2 probably due to the larger size of the pBFT2 surface groove compared to that of mBFT2.

Lipid A, as a main component of LPS, also forms H-bonds with amino acid residues on the mBFT2 groove surface (Figure 5A). The lipid A molecule contains two-fold the amount of fatty acid residues compared to PE and PC and therefore makes a significant hydrophobic contribution to mBFT2-lipid A complex formation [(Supplementary information 2 (Table 3)]. In accordance with Elhenawy et al., the lipid A molecule contains a phosphate group, but PSC may exist without the phosphate group in an aqueous medium (Elhenawy et al., 2014). PSC is composed of long chains of monosaccharide units bound together by glycosidic linkages, which form multiple H-bonds with mBFT2 amino acids (Figure 5C). These interactions may have a significant impact on polysaccharide chain-mBFT2 complex formation.

Importantly, non-ester phosphate oxygen atoms of PC/PE (Figures 4B,D), lipid A (Figure 5B), and PSC (Figure 5D) tend to form coordinate bonds with the zinc ion in the active center of mBFT2. Carbonyl oxygen atoms of PC/PE might also form such bonds with the zinc ion. According to our data, electrostatic contributions are higher for the lipid-mBFT2 complex than for the lipid-pBFT2 complex, indicating possible coordinate bond formation [Δ*Gel*_(*PE*+*pBFT*);_ Δ*Gel*_(*PC*+*pBFT*)_ < Δ*Gel*_(*PE*+*mBFT*)_; Δ*Gel*_(*PC*+*mBFT*)_] [(Supplementary information 2 (Table 3)].

### Trp fluorescence quenching assay and 1H-NMR spectroscopy of BFT-lipid complexes

**Figure 6:**
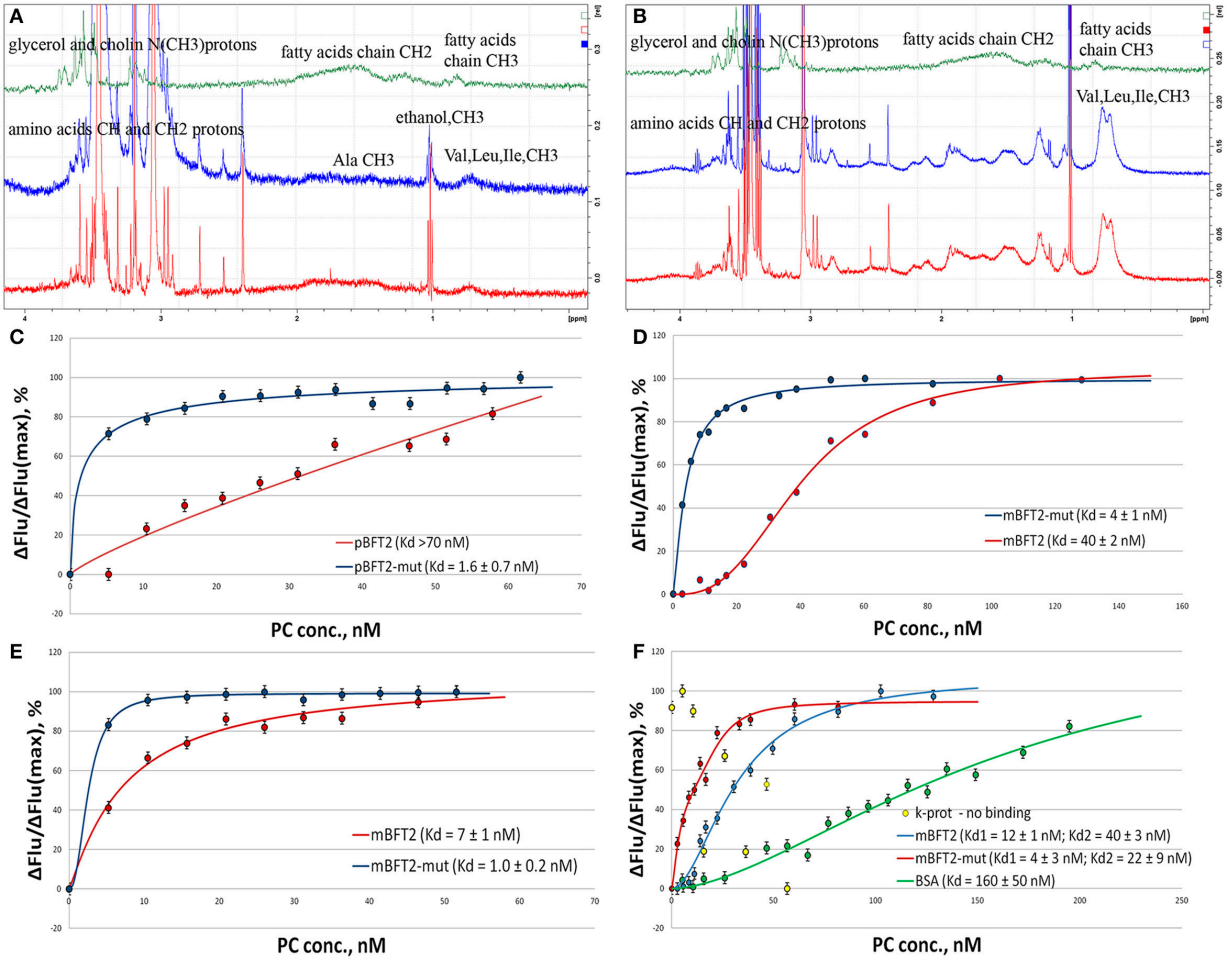
**500 MHz 1D proton**. NMR spectra of individual PC, pBFT2, and mBFT2 or their mixture **(A,B)** and fluorescence quenching assay**(C–F)**. **(A)** 500 MHz 1H-NMR spectra of PC (green), pBFT2 (blue), and their mixture (red), (0–4 ppm region). **(B)** 500 MHz 1H-NMR spectra of PC (green), mBFT2 (blue), and their mixture (red). **(C–F)** Trp fluorescence quenching. Conditions: The standard PBS buffer (**C–E**) or the low ionic strength buffer with Zn (**F**); excitation at 295 (**C,D**) or 280 nm (**E,F**) 20°C. Protein concentration was 3 nM. Error bars indicate standard deviation for three measurements.

In the above molecular modeling and docking experiments, we identified the most abundant classes of phospholipids of the *B. fragilis* membrane (PE and PC) and demonstrated their interaction with the mBFT2 and pBFT2 surfaces *in silico*. Next, the propensity of mBFT2 and pBFT2 to form complexes with PC was tested using 1H-NMR and Trp fluorescence quenching (Figure 6). Comparison of NMR spectra of PC alone and PC mixed with pBFT2/mBFT2 (Figures 6A,B) revealed changes in the chemical shifts and peak widths of the fatty acid signals (0—4 ppm), which indicated changes in PC conformation and mobility upon complex formation.

The Trp fluorescence quenching assay, a popular method for investigating lipid-protein interactions (Dua et al., 1995; Kraft et al., 2009), was chosen because BFT2 contains 4 Trp residues in its catalytic site. Proteinase K was used as a negative control, and BSA, which is known to bind lipids in the medium nanomolar range (Charbonneau and Tajmir-Riahi, 2010), was used as a positive control. Intrinsic protein fluorescence was monitored at the Trp/Tyr emission maximum upon excitation at 295 nm (Trp fluorescence, selectively, Figures 6C,D) or 280 nm (Trp and Tyr fluorescence, Figures 6E,F) in the presence of increasing concentrations of PC organized in liposomes by reverse-phase evaporation. The results suggest the formation of 1:1 protein-lipid complexes in a low ionic strength buffer and the formation of 3:1 mBFT2-lipid complexes in the standard PBS buffer. Importantly, the presence of larger aggregates with a different stoichiometry at high lipid concentrations cannot be excluded, as Trp fluorescence is likely only sensitive to initial saturation.

According to our docking models, non-ester phosphate oxygen atoms of the lipids tend to form coordinate bonds with the His-bound zinc ion in the active center of mBFT2. To test the model and the potential involvement of these coordinate bonds, we analyzed a BFT2 mutant with no His residues and, thus, no zinc ions in the active site. Both mutant forms (mBFT2-mut and pBFT2-mut) demonstrated enhanced affinity to PC, which may be attributed to their increased hydrophobicity, as the charged His residues had been replaced with polar but uncharged Tyr residues. The fact that mBFT2-mut bound with PC more efficiently than the wild-type protein argues against a major contribution by zinc to complex formation.

The Trp fluorescence quenching assay demonstrated that the most represented lipids of the *B. fragilis* cell membrane (PC and PE) form complexes with mBFT2, but to determine the lipid chemical groups capable of such interactions, NMR spectroscopy was performed. Similar to Trp fluorescence quenching assay results, constant values (K_d_) calculated for PC-mBFT2 and PE-mBFT2 complexes had slight differences, confirming the specific nature of a protein-lipid interaction. Thus, PC was used as a model of protein-lipid complex formation. We used 5 μM of each form of BFT2 (pBFT2 and mBFT2) and 0.6 μM PC in a water solution to study complex formation. Common 1D proton (with WATERGATE water suppression) (Figures 6A,B) and 2D DOSY NMR spectra of PC, BFT2 forms and mixtures were obtained. L-α-phosphatidylcholine (P3556, Sigma Aldrich) was used as the model PC. We observed that PC formed a micelle suspension in the water solution. According to the diffusion NMR spectroscopy results, the micelles consisted of a rather large number of molecules (~10 molecules per micelle). This result corresponds to the literature on this topic (Mashburn-Warren et al., 2008). The diffusion coefficients of pBFT2 and mBFT2 in water solutions were D = −8.4 log(m^2^/s) and D = −8.9 log(m^2^/s), respectively. The diffusion coefficient for PC in the water solution was D = −8.2 log(m^2^/s). The diffusion coefficients of PC and pBFT2 in the mixture were altered [D = −9.1 log(m^2^/s) and D = −8.6 log(m^2^/s), respectively] (Figure 6A) compared to the diffusion coefficients of each alone. The lipid molecules in the mixture were less mobile than in micelles in solution, and the protein molecules were also slightly less mobile in the mixture. In addition, the chemical shifts and peak widths of the fatty acid of PC in the mixture were slightly changed. Peak width changes in this case may be caused only by T_2_ relaxation time, i.e., by PC mobility or conformation change. All these changes together may be explained by phospholipids-protein complex formation. Only one type of diffusion coefficient for PC and pBFT2 was present in the mixture, indicating that only one type of protein-phospholipid complex was present in the solution. The difference between the diffusion of free pBFT and pBFT2 in complex was not large. Consequently, complex formation probably causes only a protein conformation change. The results could be interpreted as the association of small lipid particles with the protein molecule core and PC shell. The results of NMR experiments with mBFT2 were similar to those described above (Figure 6B). mBFT2 also formed a complex in mixture with PC. However, the mBFT2 molecules were also partially self-aggregated, which was shown by 2D DOSY results. There were two fractions in the mBFT2 solution with diffusion coefficients D = −8.9 log (m^2^/s) and D = −9.3 log (m^2^/s). The mobility of the protein was also lower in the mixture with PC than individually, and PC in the mixture was less mobile as well. The diffusion coefficients of PC and mBFT2 in mixture were D = −8.7 log(m^2^/s) and D = −9 log(m^2^/s), respectively. The NMR data have been deposited to UCSD Center for Computational Mass Spectrometry (MassIVE ID: MSV000080385).

### OMV-associated BFT is biologically active

**Figure 7:**
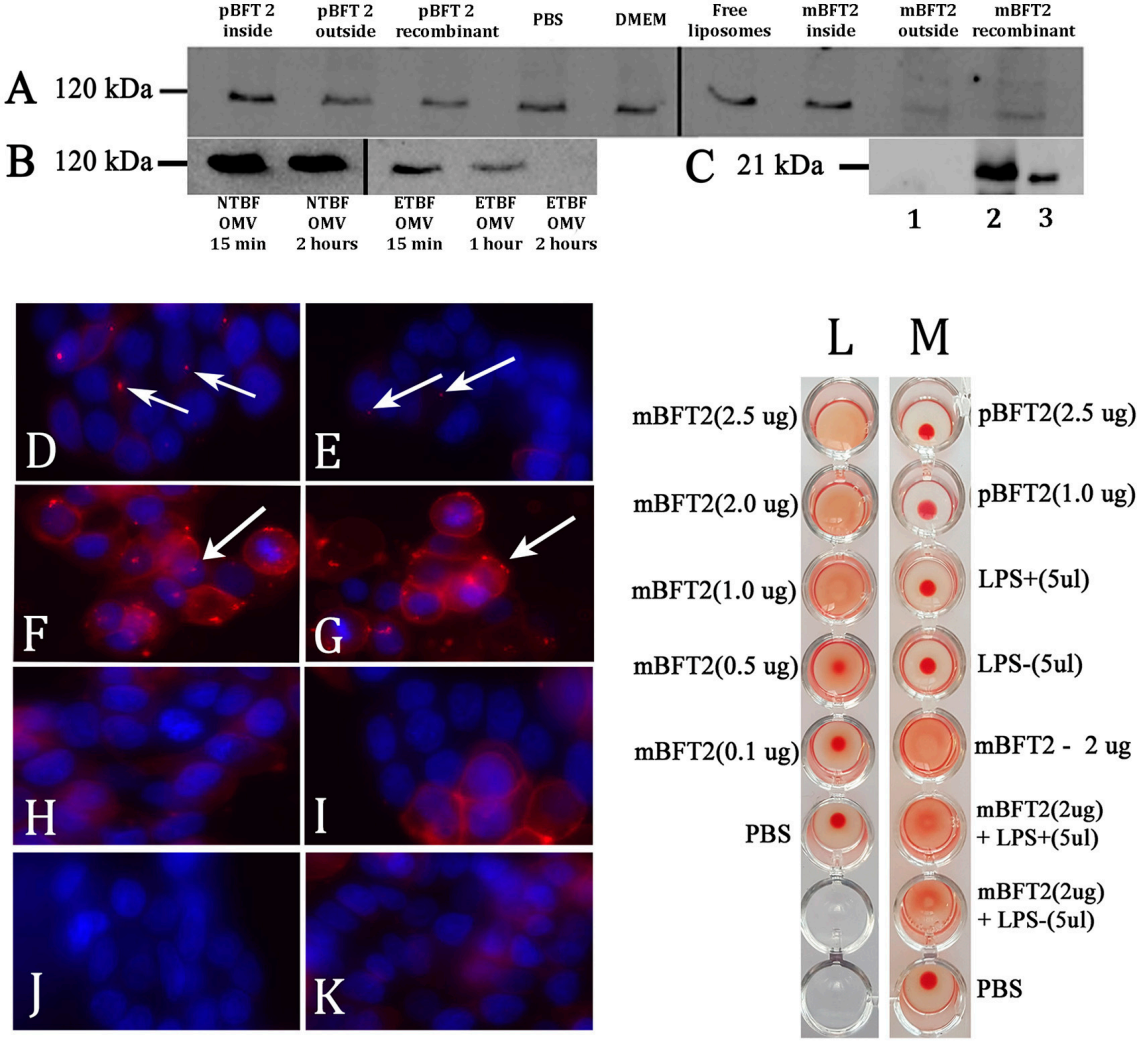
**Toxin containing liposomes and OMVs show proteolytic activity against HT-29 cell line E-cadherin and form complexes (A–K)**. Hemagglutination test **(L–M) (A)** Biological effects of the toxin to E-cadherin dependent of its localization. 12 nM pBFT2 and 14.2 nM mBFT2 encapsulated into liposome (pBFT2 inside and mBFT2 inside) or added to prepared liposomes—(pBFT2 outside and mBFT2 outside) were coincubated with HT-29 cells. Recombinant proteins were also coincubated with HT-29 cells (12 nM pBFT2–pBFT2 recombinant; 14.2 nM mBFT2–mBFT2 recombinant). PBS, DMEM and Free liposomes were used as negative controls. **(B)** Biological effect of the toxin containing OMVs to E-cadherin is time depended. 50 μg of total ETBF OMV proteins were coincubated with HT-29 cells for 15 min (ETBF OMV for 15 min), for 1 h (ETBF OMV for 1 h), for 2 h (ETBF OMV for 2 h). The same amounts of NTBF OMV proteins were coincubated with HT-29 cells for 15 min (NTBF OMV for 15 min) or for 2 h (NTBF OMV for 2 h). Extracted HT-29 cells proteins **(A,B)** were run on 10% SDS-PAGE followed by western blot with antibody against E-cadherin. **(C)** Toxin encapsulation and toxin-liposome association were examined by western blot. 14.2 nM mBFT2 with previously added prepared liposomes were dissolved in sterile culture media. After ultracentrifugation step supernatant was examined to unbound toxin—(lane 1), 14.2 nM mBFT2 encapsulated into the liposomes were treated with Proteinase K (20 ng/μl) and run on 10% SDS-PAGE followed by western blot with antibody against mBFT2–(lane 2). Recombinant protein—14.2 nM mBFT2—lane 3. **(D–K)** Complexes formation was examined by fluorescent microscopy. 3 μg OMVs, isolated from ETBF-**(D)** and NTBF-**(E)**; 12 nM pBFT2-**(F)** and 14.2 nM mBFT2-**(G)** were added to 100 μl prepared liposomes; 14.2 nM mBFT2-**(H)** and 12 nM pBFT2-**(I)** were encapsulated into the liposomes. **(J)**-10 μl VybrantDiI were added to PBS and centrifuged at 100,000 g. **(K)**-100 μl liposomes. All samples **(D–I, K)** were labeled with VybrantDiI (red) and coincubated with HT-29 cells for 1 h. Nuclei were stained with DAPI (blue). Arrows indicate protein-lipid complexes located on cells surfaces. **(L)**-Hemagglutination activity of mBFT2 depends on toxin concentration. **(M)**-Hemagglutination activity of mBFT2 and pBFT2 in a presence of LPS.

E-cadherin is a 120-kDa type I transmembrane protein essential to the intercellular adhesion of adjacent epithelial cells, which are the primary known substrate for BFT protease activity (Remacle et al., 2014). Given that mBFT2 was associated with OMVs, vesicle treatment should promote E-cadherin degradation in HT-29 cells. Using western blot analysis (Figure 7B), we observed time-dependent degradation of the 120-kDa subunit E-cadherin and its complete cleavage after 2 h of treatment with ETBF OMV in HT-29 cells. To confirm the effect of mBFT2-containing OMVs of ETBF on E-cadherin degradation, we used NTBF OMV at the same time points, and there was no effect.

The effect of toxins on E-cadherin could be dependent on mBFT2 OMV localization. BFT2 can be located inside the vesicles, in the vesicle membrane, or on the OMV surface. We produced two types of liposomes that were reverse-phase constructed with PC. In the first case, the toxin was encapsulated into the liposomes by a reverse-phase evaporation method, while in the second case, mBFT2 was added to the prepared liposomes. We verified the encapsulation of BFT2 into liposomes and analyzed whether unbound BFT2 was present in the medium by western blot (Figure 7C). We found that all of the added BFT2 was associated with liposomes, as we did not observe free BFT2 in the medium after centrifugation. Two types of liposomes with mBFT2 and pBFT2 were used for E-cadherin degradation experiments.

We observed complete E-cadherin degradation in HT-29 cells after 2 h of treatment with liposomes with added mBFT2 (Figure 7A). The same effect was shown using mBFT2 without liposomes. There was no E-cadherin cleavage when HT-29 cells were incubated with mBFT2 encapsulated into the liposomes. As expected, we did not observe any effect of pBFT2 on E-cadherin.

As described previously, OMVs can be used for virulence factor delivery to host cells. OMVs isolated from ETBF (Figure 7D) and NTBF (Figure 7E) were labeled with the lipophilic dye DiI, and two forms of the toxin [mBFT2 (Figure 7F) and pBFT2 (Figure 7G)] were independently added to the prepared liposomes and then incubated with HT-29 cells for 1 h. We also tested two forms of the toxin [mBFT2 (Figure 7H) and pBFT2 (Figure 7I)] encapsulated in liposomes for their ability to form complexes. As a control, we used free DiI-labeled liposomes that were also incubated with HT-29 cells for 1 h (Figure 7J). Moreover, to reduce possible false positive cell coloring, we dissolved 10 μl of stock DiI solution in PBS and centrifuged it at 100,000 × g; the resulting sediment dissolved in PBS was used for HT-29 cell labeling (Figure 7K). In agreement with our previous results, mBFT2 and pBFT2 added to the prepared liposomes formed complexes located on the cell surface (Figure 7F,G, **protein-lipid complexes indicated by arrows**). We observed ETBF OMVs and NTBF OMVs located on the cell surface, confirming that OMVs are used for protein delivery. Additionally, the relatively few NTBF OMVs located on the cell surface compared with ETBF OMVs may indicate that the toxin facilitates adherence between OMVs and cell membranes. We did not observe any labeled liposomes with encapsulated toxins on the cell surface, confirming the role of the toxin in complex formation.

### Hemagglutination test

Since pBFT2 and mBFT2 tend to form H-bonds and coordinate bonds with membrane components, including LPS, we examined the hemagglutination and lectin activity for both types of the toxin. First, increasing concentrations (0.1–2.5 μg) of mBFT2 were tested for the ability to induce hemagglutination. We detected erythrocyte hemagglutination with mBFT2 at concentrations of 2.5 and 2 μg (Figures 7L,M). pBFT2 did not demonstrate hemagglutination activity. We also identified inhibition of hemagglutination when mBFT2 was premixed with LPS isolated from ETBF and NTBF. LPS alone did not demonstrate hemagglutination activity (Figure 7M).

## Discussion

OMVs are considered to be one of the types of bacterial secretion system. Moreover, this secretion mechanism is well known in *B. fragilis* (Patrick et al., 1996; Elhenawy et al., 2014). Recently it has been described that *B. fragilis* contains T6SS, which is likely a source of numerous novel effector and immunity proteins (Coyne et al., 2016). But there is no evidence that this could be the mechanism for toxin secretion. In our study we hypotized that OMVs can be utilized for *B. fragilis* toxin secretion.

In our experiments, we demonstrated that both strains (ETBF and NTBF) of *B. fragilis* produce vesicles, and we detected mBFT2 in vesicles by western blot assay, suggesting that OMVs could be the mechanism of BFT secretion. As expected, microscopy assay results were in agreement with cells fractions and OMVs western blot results. We observed labeled antibodies against BFT2 in the periplasm, in membrane and in the vesicle membranes. Moreover, pBFT2 was detected primarily in membrane fraction but was not detected in OMVs suggesting possible toxin maturation in cells membrane. An NTBF cell fraction and OMVs isolated from NTBF culture were used as negative controls in all experiments.

The main visual biological effect of toxin exposure is that mBFT2 affects zonula adherens by cleaving E-cadherin. We used OMVs isolated from ETBF and NTBF to evaluate their biological activity against E-cadherin. As expected, we detected time-dependent E-cadherin degradation upon ETBF OMV treatment, but no degradation with NTBF OMV treatment, confirming that the toxin is active and associated with OMVs.

According to a recent publication, there is a special mechanism of OMV protein sorting. It was shown in *Campylobacter jejuni* that CDT toxin is not associated with membranes and is localized on the inner surface of the vesicles. This result was confirmed by OMV protease treatment, during which the toxin was not degraded because it was inside the vesicles (Lindmark et al., 2009). We used Proteinase K (20 ng/μl) treatment to determine possible toxin localization by western blot analysis and found that BFT2 was fully degraded within 10 min of Proteinase K treatment. These results indicate that the toxin is located on the OMV surface. To confirm surface-located toxin activity against E-cadherin, we prepared several types of liposomes with different localizations of the mBFT2. pBFT2 was used as a negative control because the immature toxin form does not contribute to E-cadherin degradation (Wu et al., 1998). In HT-29 cells treated with all liposome-toxin preparations, we observed E-cadherin degradation only when the toxin was added to the previously prepared liposomes, indicating that OMV-associated BFT2 is located on OMV surface. We observed toxin-liposome complexes labeled with the lipophilic dye DiI on the surface of HT-29 cells. Complex formation occurred only in the presence of toxin, which acted as an adhesive element. As expected, both types of OMV (isolated from ETBF and NTBF) were found on the HT-29 cell surface. Most likely, the ETBF and NTBF vesicle membranes contain adhesion proteins that facilitate OMV binding to the cell surface followed by internalization.

BFT could interact with a membrane protein receptor sensitive to depletion of membrane cholesterol (Wu et al., 2006). In this study, we hypothesized that BFTs interact with OMV membrane components, including phospholipids and LPS before binding with the potential receptor. First, we identified the *B. fragilis* membrane lipids and LPS using mass spectrometry. As expected, the most represented ions in the spectrum belonged to PE and PC classes, as they are prevalent lipid components of gram-negative bacteria membranes (Epand et al., 2007). Furthermore, we identified the lipid A and polysaccharide chain structures of *B. fragilis* LPS.

These results were further used for modeling and docking experiments. The mass spectrometric analysis results we obtained completely corresponded to data previously reported by Elhenawy et al. (2014). It should be noted that Elhenawy et al. previously characterized the same structure of lipid A and showed no difference between bacterial outer membrane and OMV lipid A structure. We identified that *B. fragilis* LPS consisted of a poly- and oligosaccharide region composed by monogalactosyl and -glucosyl units (up to 8 carbohydrate units in the chain) linked to lipid A-disaccharide.

We used the identified phospholipids, lipid A, and polysaccharide chain structures to model their interaction with mBFT2 and pBFT2. Polysaccharide chain and lipid A showed comparable binding energy values in docking with mBFT2. As expected, we observed the formation of multiple H-bonds between monosaccharide units of polysaccharide chain and amino acid residues of mBFT2, but an unusual effect was the coordinate bond formation between the oxygen of phosphate groups and zinc ion. This interaction is similar to the formation of non-covalent bonds in lectins, which have special binding sites for polysaccharides and form coordinate bonds with metal ions (Sharon, 1987; Abhilash et al., 2013). However, lectins are characterized by non-covalent interaction with sugars, not with lipids. The docking procedure for pBFT2 and LPS components was not performed because we did not expect the immature form of the protein to bind to LPS.

Despite the fact that mBFT2 is a soluble protease, we demonstrated, through the docking modeling that both mBFT2 and pBFT2 might hydrophobically interact with phospholipids. Carbonyl oxygen atoms of PC/PE (as it was shown for LPS components) formed coordinate bonds with the zinc ion located in the active center of mBFT2 after its processing. By diffusion NMR spectroscopy and Trp fluorescence quenching assays, we evaluated the formation of pBFT2-lipid and mBFT2-lipid complexes. NMR data indicated that the fatty acid residues participate directly in these interactions. The mutant protein mBFT2-mut, which does not contain a zinc ion in the active site, demonstrated enhanced affinity for PC, which argues against a major role for the coordinate bonds. We also observed enhanced affinity of the mutant form of pBFT2 for PC, which could result from the conformational alterations caused by the mutation or somewhat increased hydrophobicity.

High lipid-protein tropism was also shown in cell culture experiments. The formation of mBFT2-lipid complexes associated with the cell surface was demonstrated by microscopic assay. It was also shown that labeled vesicles were located on the cell surface. Our microscopy results confirmed previous experiments reported by Sears et al. where labeled recombinant toxin was also located on the cell surface (Wu et al., 2006).

**Figure 8:**
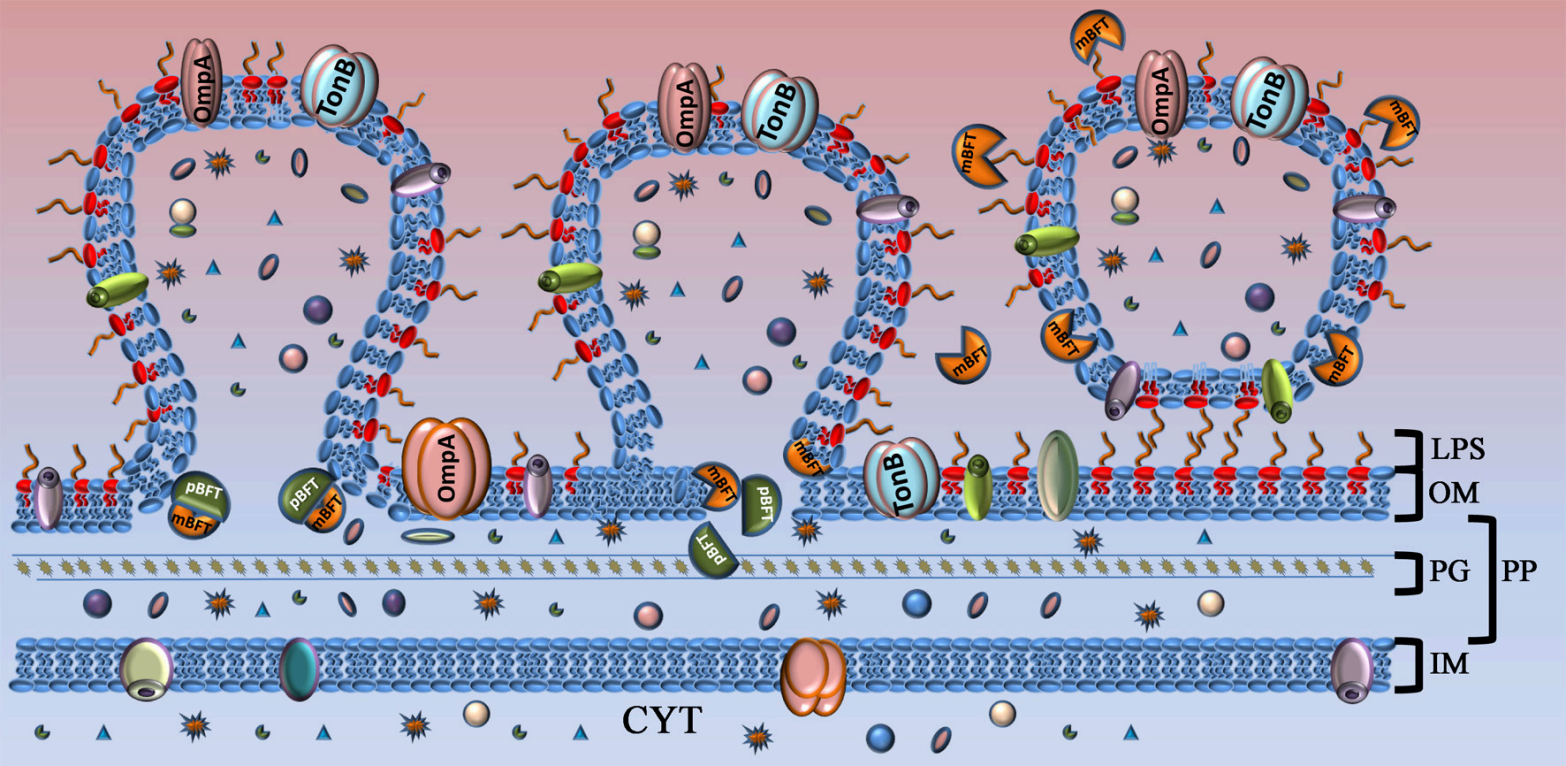
**Model of toxin capture during vesicle formation**. Toxin is shown as complex of two subunits. Part of the toxin that will be processed (pBFT) and degraded is colored in dark green. Active part of the toxin (mBFT) colored in orange. OM, outer membrane; LPS, lipopolysaccharide; PG, peptidoglycan; PP, periplasm; IM, inner membrane; CYT, cytoplasma. At the beginning of vesicle formation, the major part of the toxin is not processed and could be found in membrane. During vesicle formation toxin could be processed by *B. fragilis* cysteine protease. Free grooves, located on the protein surface, formed during toxin processing interact with fatty acid residues of outer membrane lipids by hydrophobic interaction. Toxin promoted to the OMV surface where it interact with LPS. Incorporated or tightly binding toxin delivered to the target cell by OMVs.

Based on the obtained results, we propose a mechanism of toxin secretion (Figure 8). pBFT2 interacts with cellular membrane lipids and accumulates in the inner membrane. In periplasm possibly it undergoes processing via proteolytic cleavage by a *B. fragilis* cysteine protease, forming the active version of the toxin (mBFT2) (Herrou et al., 2016). Oxygen atoms of lipid phosphate groups located on the inner surface of the outer membrane bilayer interact with the active center of mBFT2, simultaneously forming coordinate bonds with the zinc ion. Thermal motion of the protein leads to a local stretching of the lipid, facilitating the cessation of hydrophobic interactions with its membrane pair. Fatty acid tails of lipids tend to occupy the free grooves in mBFT2, resulting in the promotion of mBFT2 outside the outer membrane. At this point, vesicle formation could occur, and the toxin incorporated into the OMV membrane will be delivered to the host cell. However, the proposed model of toxin promotion through the membrane does not exclude the possibility of potential secretion system involvement.

The protein could also be promoted to the OMV surface, forming new, stronger electrostatic and hydrogen bonds with LPS components and disrupting previous focal and hydrophobic bonds between the lipid and protein. Further protein motion becomes impossible due to significant hydrogen bond formation between the LPS carbohydrate moiety and the toxin. The same protein-carbohydrate communication was demonstrated previously in numerous studies for lectins (Soult et al., 2014). Lectins and carbohydrates are linked by a number of relatively weak interactions that ensure specificity. The interactions between one cell surface with carbohydrates and another with lectins resemble the action of Velcro in that each interaction is relatively weak but the composite is strong. Today, the adhesins and toxins produced by *Vibrio cholerae, Shigella dysenteriae*, and *Bordetella pertussis* are the most significant microbial lectins (Sandros et al., 1994). To confirm the potential lectin activity of mBFT2, we tested the ability of the toxin to induce hemagglutination. Here, we show for the first time that mBFT2 has hemagglutination activity. However, lectin activity was determined by the ability of LPS to inhibit hemagglutination. We premixed mBFT2 with LPS isolated from ETBF and NTBF and demonstrated alteration of the hemagglutination activity of mBFT2. Erythrocytes tended to form pellets when LPS had been mixed with mBFT2. Of note, pBFT2 did not display hemagglutination activity. We suggest that pBFT2 does not have the ability to induce hemagglutination but acquires this property during processing. Two forms of the toxin that have different hemagglutination properties might also exhibit different interaction with lipids. Thus, we demonstrated that mBFT2 has strong lectin activity that could be necessary for its biological functions.

We believe that the strong lectin-like protein-membrane association identified here could be used in two potential mechanisms of toxin-host cell interaction. Toxin-lipid or toxin-LPS association leading to the local toxin concentration increasing on the surface of target cells may increase contact of the toxin with a corresponding substrate or receptor. Moreover, toxin associated with vesicles could be taken up via endocytosis by eukaryotic cells or undergo a membrane flip-flop mechanism and persist in the intracellular environment.

## Materials and methods

### Bacterial strains and growth conditions

Enteropathogenic *B. fragilis* BOB25 (Nikitina et al., 2015) and non-toxigenic *B. fragilis* 323-J-86 (clinical isolates kindly provided from the Federal Research and Clinical Centre of Physical-Chemical Medicine Federal Medical Biological Agency, Moscow, Russia) were grown on blood agar plates containing either 5% defibrinated horse blood or brain heart infusion broth supplemented with hemin (5 g/ml) under anaerobic conditions.

### Cell line medium and culture conditions

Human colonic epithelial cells (HT-29) obtained from the American Type Culture Collection (ATCC Number HTB-38) were seeded in 25 cm^2^ flasks at 37°C under 5% CO_2_ in DMEM (Life Technologies, USA) containing 10% fetal bovine serum (FBS, Life Technologies, USA) and 2 mM GlutaMax (Life Technologies, USA).

### Recombinant proteins and antibodies

Immature and mature forms of recombinant toxins (mBFT2, pBFT2) were prepared as described previously (Kharlampieva et al., 2015). The mutant forms of BFT (mBFT2-mut, pBFT2-mut) were obtained by site-directed mutagenesis, where zinc-chelating histidine residues were mutated to tyrosine residues (H348Y, H352Y, and H358Y). Detailed information about how the mutant forms of the toxin have been described previously (Kharlampieva et al., 2015).

For BFT2-specific antibody preparation, 100 μg recombinant pBFT2 in 1x PBS was injected to rabbits four times at intervals of 3–4 weeks. Fourteen days after the final injection, 15 ml blood was collected. Immunoglobulin fraction separation from the serum was performed with protein A-sepharose.

### OMV purification

Overnight-cultured *B. fragilis* (ETBF and NTBF) strains were centrifuged at 4500 × g at 4°C. To remove residual cells, the supernatant was filtered using a 0.45-μm pore membrane (Millex GV; Millipore). The filtrate was subjected to ultracentrifugation at 100,000 × g for 2 h (Optima L-90K ultracentrifuge; Beckman Coulter). The supernatant was discarded, and the pellet was washed with sterile PBS and filtered through a 0.2-μm pore polyvinylidene difluoride (PVDF) membrane (Millex GV; Millipore). The ultracentrifugation step was repeated. The vesicle pellet was resuspended in 150 mM NaCl (pH 6.5). Protein concentration was quantified using a 2D-quant kit (GE Healthcare Life Sciences).

### Cell fractionation

Cell fractionation was performed as described by Lindmark et al. (2009). Precipitated proteins from cell fractions were collected by centrifugation at 12,000 × g, washed with acetone, dried and dissolved in Laemmli sample buffer. Protein concentration was quantified using a 2D-quant kit (GE Healthcare Life Sciences). In total, 40–60 μg each extract sample was used for SDS-PAGE and western blot assay.

### Cells fractions LC-MS/MS analysis

Detail information about **SDS PAGE** and **In-gel trypsin digestion** of cell fractions proteins can be found in Supplemental information 2. LC-MS/MS analysis of tryptic peptides was carried out using Ultimate-3000 HPLC system (Thermo Scientific) coupled to a maXis qTOF after HDC-cell upgrade (Bruker) with a nano-electrospray source. Chromatographic separation of peptides was performed on a C-18 reversed phase column (Zorbax 300SB-C18, 150 mm x 75 um, particle diameter 3.5 um, Agilent). Gradient parameters were as follows: 5–35% acetonitrile in aqueous 0.1% (v/v) formic acid, duration 120 min, column flow 0.3 ul/min. Positive MS and MS/MS spectra were aquired using AutoMS/MS mode (capillary voltage 1700, curtain gas flow 4 l/min, curtain gas temperature 170 C, spectra rate 10 Hz, 4 precursors, m/z range 50–2200, active exclusion after 2 spectra, release after 0.5 min). Detail information about **Search Database Creation** and **Proteins and Peptides Identification** can be found in Supplemental information 2.

### SDS-PAGE and western blot analysis

The isolated OMVs and different cellular extracts were mixed with Laemmli sample buffer (1:1) containing CHAPS and separated by SDS-PAGE. A total of 40 μg each (NTBF and ETBF) OMV sample and 40–60 μg periplasmic, cytoplasmic, and membrane fractions were used for SDS-PAGE and subsequent western blot analysis. We used BFT2-specific antibodies and horseradish peroxidase-linked anti-rabbit IgG (from sheep, dilution 1:10,000, GE Healthcare, USA). The membranes were processed with ECL Plus western blot detection reagents (GE Healthcare, USA) according to the manufacturer's guidelines. The signals were detected on a ChemiDoc MP (BioRad, USA).

### Electron microscopy and immunogold labeling

Ultrathin sections of ETBF and NTBF cells were prepared as previously described (Farquhar, 1956). A volume of 5 μl of each OMV samples was negatively stained with 2% (wt/vol) uranyl acetate for 3 min and examined using a Zeiss Libra 120 electron microscope (Zeiss, Germany).

For immunogold labeling, cells were fixed by the addition of formaldehyde and glutaraldehyde to final concentrations of 4 and 0.1–0.2%, respectively, dehydrated with ethanol in increasing concentrations (70, 96%) and embedded in LR-White resin (Polyscience, INC, USA). To visualize the distribution of the BFT protein in *B. fragilis* cells, BFT2-specific antibodies diluted 1:50 in PBS (1×) and conjugates of protein A with 15 nm colloid gold particles (Aurion, The Netherlands) were used. OMVs were adsorbed onto carbon-coated nickel grids for 2 min and coincubated with labels, coupled with antibodies against BFT, and then negatively stained. Immunogold labeled samples were examined using a Zeiss Libra 120 electron microscope (Zeiss, Germany).

### Lipid extraction

For lipid extraction, 1 ml liquid culture (ETBF and NTBF) was used. Samples were incubated at –77°C for 15 min, then at room temperature for 3 min and centrifuged at 10,000 × g for 20 min. The resulting cell pellets were used for lipid extraction by a modified Folch protocol (Folch et al., 1957). LPS was isolated using the Tri-Reagent method described by Yi and Hackett (2000).

### Shot-gun lipidomics

Identification of extracted membrane lipids and LPS was performed using a quadrupole time-of-flight tandem mass spectrometer (Q-TOF Maxis, Bruker Daltonics, Germany) with an updated collision cell for electrospray ionization source. Metabolite identification was confirmed by the fragmentation of the detected parent ions. External phospholipid standards (choline, phosphatidylcholine, phosphatidylserine, phosphatidylethanolamine, phosphatidylglycerol, phosphatidic acid, and tetracycline; Sigma Aldrich) were used to optimize the MS/MS-acquisition conditions. We used the following databases for lipid identification: LIPID MAPS database (LIPID MAPS Lipidomics Gateway, a free resource sponsored by the National Institute of General Medical Sciences, USA; http://www.lipidmaps.org), Byrdwell G. Resources for Lipid Analysis in the twenty-first Century (http://www.byrdwell.com), Metlin (Scrips Center for Mass Spectrometry, USA; https://masspec.scripps.edu) and Galactosylceramides and Glucosylceramides (Cerebrosides) (http://www.lipidhome.co.uk/).

### Modeling and docking

No BFT2 crystal structures have been reported thus far, so the atom coordinates for creating 3D models of this protein were taken from the XRD-based structure of its homolog BFT3 (PDB: 3p24 http://www.rcsb.org/pdb/explore/explore.do?structureId=3p24) by substituting several amino acid residues (see Supplemental information 2). The 3D models of the targets (PE, PC, Lipid A, and polysaccharide chain of LPS) were created with the Molsoft ICM version 3.8–3 application (Wesson and Eisenberg, 1992; Abagyan et al., 1994; Totrov and Abagyan, 2001a,b). Detailed information about the modeling and docking procedure can be found in Supplemental information 2.

### Trp fluorescence quenching assay

Intrinsic fluorescence of the Trp and Tyr residues was measured after the addition of different amounts of vesicles constructed from PC to 3 nM mBFT, pBFT2, mBFT2, pBFT-mut, mBFT-mut, Proteinase K, or BSA solutions in the standard PBS buffer (Merck) or a low ionic strength buffer (1 nM ZnSO_4_ in 1:50 diluted PBS). Each protein solution was mixed gently after the addition of the PC vesicles and stored at room temperature for 1–2 min prior to measurements. Fluorescence emission spectra were registered at 20°C using a Chirascan spectrometer (Applied Photophysics) equipped with a thermostatically controlled cuvette holder with slit widths of 4 and 6 nm upon excitation at 280 or 295 nm. Light scattering by liposomes was taken into account. The quenching curves were fitted with equation (1)

(1) $$Y=\frac{Y_{\max}\times X^{n}}{X^{n}+K_{d}^{n}}$$

where Y is fluorescence quenching and *n* is the Hill coefficient (*n* = 1 ± 0.3 unless otherwise specified).

Fitting was performed using DataFit 9 software.

### NMR analysis of protein-lipid complexes

The samples for NMR spectroscopy [500 μg mBFT2, 500 μg phosphatidylcholine (Sigma Aldrich, USA)] were dissolved in water (100 μl D_2_O was added to each sample for the lock signal stabilization) and phosphate buffer (pH 7.4). All spectra were obtained on a Bruker Advance III 500 MHz NMR spectrometer (Bruker, USA) equipped with a Prodigy TCI cryogenic triple-channel probe. The sample temperature was kept at 300 K during the experiments. 2D DOSY (the stimulated echo pulse sequence with bipolar gradient pulses was used) and common 1D proton spectra were measured for the study of protein-lipid complex formation (Chou et al., 2004; Balayssac et al., 2009). The water peak in all experiments was suppressed by WATERGATE pulse sequence with five pairs of symmetric gradients (125 ms delay for binomial water suppression and 200 ms delay for gradient recovery). The NMR data processing and analysis were performed using Bruker TopSpin v.3.2 NMR.

### Preparation of liposomes by reverse-phase (REV) evaporation

To prepare liposomes, 30 μl phosphatidylcholine or phosphatidylethanolamine in chloroform solution (100 mg/ml) and 300 μl PBS with 1 ml diethyl ether were added to a 50 ml round-bottom flask with a long extension neck, and the solvent was removed under reduced pressure by a rotary evaporator. Prepared liposomes were dissolved in 200 μl PBS.

### Cell culture experiments

To examine the biological activity of BFT2-containing OMVs, HT-29 cells were co-incubated for 2 h with 40–60 μg total OMV proteins isolated from ETBF and NTBF. After co-incubation, cells were washed with 1x PBS buffer several times and mixed with 50 μl 1x Laemmli sample buffer containing CHAPS. To detect E-cadherin cleavage, western blot analysis with E-cadherin monoclonal mouse antibody (dilution 1:1000, Invitrogen, USA) and horseradish peroxidase-linked anti-mouse IgG (from sheep, dilution 1:10,000, GE Healthcare, USA) was performed. The membranes were processed with ECL Plus western blot detection reagents (GE Healthcare, USA) according to the manufacturer's guidelines. The signals were detected on a ChemiDoc MP (BioRad, USA).

To determine mBFT2 localization (on the outer or inner surface of the OMV membrane) essential for biological activity against E-cadherin, two different types of liposomes were obtained by reverse-phase evaporation. For encapsulated toxin preparation, 14.2 ng mBFT2 and 12 nM pBFT2 were encapsulated into 30 μl (100 mg/ml) liposomes by reverse-phase evaporation method, described earlier (“Material and Methods”—“Preparation of liposomes by reverse-phase (REV) evaporation”). Proteolysis of unencapsulated mBFT2 was performed using Proteinase K (20 ng/μl) treatment. Toxin encapsulation was verified by western blot with BFT-specific antibodies. For the toxin added to the prepared liposomes, 14.2 ng mBFT2 and 12 nM pBFT2 were added to 30 μl previously prepared liposomes (100 mg/ml), and the mBFT2-liposome mix was purified by one ultracentrifugation step at 100,000 × g for 1 h. The amount of unbound mBFT2 in the medium was determined by western blot with BFT2-specific antibodies. Both types of liposomes were co-incubated with HT-29 cells for 1 h. After incubation, cells were washed several times with 1 × PBS and mixed with 50 μl 1x Laemmli sample buffer containing CHAPS. To detect E-cadherin cleavage, western blot analysis with anti E-cadherin antibody was performed.

For visual assessment of protein-lipid complexes (mBFT2/pBFT2-liposomes) and OMV fixed on the cell surface, fluorescence microscopy was performed. Briefly, isolated OMVs of NTBF and ETBF (40 μg each) and 50 μl prepared liposomes (100 mg/ml) were separately labeled with Vybrant DiI in a 30 min incubation and purified again by one step of ultracentrifugation at 100,000 × g for 1 h. Labeled OMVs, liposomes alone, prepared liposomes with added mBFT2 or pBFT2 and encapsulated toxin preparations were co-incubated with HT-29 cells for 1 h. The cells were then washed ~three times, incubated with DAPI for nuclei labeling and observed with a Nikon Eclipse E800 microscope (Nikon, Japan) or an Olympus Live Cell Imaging System (Olympus IX51, Japan).

### Hemagglutination assay

A hemagglutination assay was performed using red blood cells (RBCs) from humans. Blood was provided by volunteer donors (Lewis O^α−β+^). Written informed consent from participants was obtained and approved by the ethical committee of the Federal Research and Clinical Centre of Physical-Chemical Medicine Federal Medical Biological Agency, Moscow, Russia (approval number 1a/2016). Heparinized whole human blood erythrocytes were washed three times by centrifugation at 200 × g and resuspended to 1.5% packed cell volume in 1 × PBS. mBFT2 in increasing concentrations (0.1–2.5 μg), 5 μl LPS (isolated from 20 ml ETBF and 20 ml NTBF) and pBFT (2.5 and 1 μg) were independently mixed with the erythrocyte suspension in a round-bottomed 96-well dish and agitated by hand. Separately, mBFT2 (2 μg) was premixed with ETBF LPS or NTBF LPS, and then prepared solutions were mixed with the erythrocyte suspension in a round-bottomed 96-well dish and agitated by hand. A positive result was recorded if hemagglutination occurred within 10 min.

## Author contributions

NZ, VT, AAV, AMV, and VP designed and performed experiments, analyzed data, and wrote the paper; DR, IV designed and performed experiments, FL, EG, DK, VM, and VL performed experiments; VG supervised the project.

## Funding

This research was supported by RSF grant 14-24-00159 and RSF 14-25-00013.

### Conflict of interest statement

The authors declare that the research was conducted in the absence of any commercial or financial relationships that could be construed as a potential conflict of interest.
