# Corrigendum: Interaction of *Bacteroides fragilis* Toxin with Outer Membrane Vesicles Reveals New Mechanism of Its Secretion and Delivery

**DOI:** 10.3389/fcimb.2017.00308

**Published:** 2017-06-30

**Authors:** Natalya B. Zakharzhevskaya, Vladimir B. Tsvetkov, Anna A. Vanyushkina, Anna M. Varizhuk, Daria V. Rakitina, Victor V. Podgorsky, Innokentii E. Vishnyakov, Daria D. Kharlampieva, Valentin A. Manuvera, Fedor V. Lisitsyn, Elena A. Gushina, Vassili N. Lazarev, Vadim M. Govorun

**Affiliations:** ^1^Federal Research and Clinical Centre of Physical-Chemical Medicine Federal Medical Biological AgencyMoscow, Russia; ^2^Department of Polyelectrolytes and Surface-Active Polymers, Topchiev Institute of Petrochemical SynthesisMoscow, Russia; ^3^Department of Molecular Virology, FSBI Research Institute of Influenza, Ministry of Health of the Russian FederationSaint Petersburg, Russia; ^4^Lab of Genome Structural Organization, Institute of Cytology, Russian Academy of SciencesSaint Petersburg, Russia; ^5^Institute of Nanobiotechnologies, Peter the Great St. Petersburg Polytechnic UniversitySaint Petersburg, Russia; ^6^N.F. Gamalei Federal Research Centre for Epidemiology and Microbiology, Ministry of Health Russian FederationMoscow, Russia; ^7^Lab of Systems Biology, Moscow Institute of Physics and TechnologyDolgoprudny, Russia; ^8^Department of Proteomics, Shemyakin-Ovchinnikov Institute of Bioorganic Chemistry of the Russian Academy of SciencesMoscow, Russia

**Keywords:** toxin delivery, lipid protein interactions, shot gun lipidomics, electron microscopy, fluorescence quenching, NMR

There is an error in the Funding statement. The correct number for the Russian Science Foundation is 16-15-00258. The corrected Funding statement appears below. The authors apologize for this error and state that this does not change the scientific conclusions of the article in any way.

## Conflict of interest statement

The authors declare that the research was conducted in the absence of any commercial or financial relationships that could be construed as a potential conflict of interest.

