# Supplementary material for: Interaction of Bacteroides fragilis Toxin with Outer Membrane Vesicles Reveals New Mechanism of Its Secretion and Delivery
Source: Front Cell Infect Microbiol. 2017 Jan 17;7:2. doi: 10.3389/fcimb.2017.00002 (PMC5240029; doi:10.3389/fcimb.2017.00002)
Supplement: Supplementary file 2 [file DataSheet2.DOCX]

SUPPLEMENTARY INFORMATION 2

Supplementary Table 1 The full list of the identified ions in membrane lipids preparation performed using quadrupole time-of-flight tandem mass spectrometer (Q-TOF Maxis, Bruker Daltonics, Germany) with an updated collision cell for electrospray ionization source.

| **Compound name (Positive mode)** | **m/z** | **Δppm** | **Adduct** |
| --- | --- | --- | --- |
| PC fragment or C27:1n-7 | 409,168 | 0 | [M+H]+ |
| (21:0/16:0) fragment | 419,282 | 3 | [M+2Na]2+ |
| Tetracyclin | 445,166 | 1 | [M+H]+ |
| Palmitoleyl palmitoleate | 459,470 | 28 | [M+H-H2O]+ |
| PC(15:0/0:0) | 464,310 | 2 | [M+H-H2O]+ |
| PC(17:1(10Z)/0:0) | 507,333 | 0 | [M+H]+ |
| DG(15:0/16:1) | 517,470 | 14 | [M+H]+ |
| Heneicosanyl oleate WE(21:0/16:1) | 531,538 | 0 | [M+H-H2O]+ |
| DG(O-16:0/18:1) | 545,545 | 0 | [M+H-2H2O]+ |
| PC(16:0/3:0) | 551,360 | 6 | [M+H]+ |
| PC(O-16:0/O-5:1) | 595,388 | 11 | [M+2Na-H]+ |
| PA(16:1(9Z)/12:1) | 611,356 | 0 | [M+Na]+ |
| PE(17:0/12:0) | 614,460 | 7 | [M+H-2H2O]+ |
| PE(12:0/18:3) | 640,418 | 6 | [M+H-H2O]+ |
| PE(14:1(9Z)/16:1) | 660,453 | 0 | [M+H]+ |
| PE(13:0/16:0) | 672,455 | 3 | [M+Na]+ |
| PA(16:0/17:0) | 664,492 | 1 | [M+H]+ |
| PA(22:0/12:0) | 677,515 | 0 | [M+H]+ |
| PE(16:0/14:0) | 686,479 | 4 | [M+H]+ |
| PE(15:0/16:0) | 700,491 | 2 | [M+Na]+ |
| PE-Cer(d16:2/20:1) | 701,523 | 0 | [M+H]+ |
| PS(O-16:0/17:1) | 716,542 | 0 | [M+H-H2O]+ |
| TG(16:0/16:0/16:0) | 845,6799 | 23 | [M+K]+ |
| **Compound name (Negative mode)** | **m/z** | **Δppm** | **Adduct** |
| Cer(d15:1/18:1) | 566,478 | 2 | [M+CH3COO]- |
| Cer(t18:0/16:0) | 590,491 | 0 | [M+Cl]- |
| DG(15:0/22:3/0:0) | 653,510 | 4 | [M+Na-2H]- |
| PC(12:0/15:0) | 662,477 | 0 | [M-H]- |
| PA(15:1/19:1) | 671,453 | 19 | [M-H]- |
| PE(15:0/16:0) | 676,484/677,472 | 12 | [M-H]- |
| PG(15:0/16:0) | 689,486 | 15 | [M-H2O-H]- |
| PE-Cer(17:1/15:0) | 692,488 | 4 | [M+F]- |
| PE(P-14/15:0) | 692,488 | 0 | [M+FA-H]- |
| PS(O-16:0/O-16:0) | 706,554 | 21 | [M-H]- |
| PE(15:0/18:3) | 720,463 | 6 | [M+Na-2H]- |
| PE(15:0/18:3) | 735,481 | 6 | [M+Na-2H]- |
| CL(20:3/20:3/18:2/18:2) | 749,500 | 6 | [M-2H]2- |

Supplementary Table 2 The full list of the identified ions in LPS preparation performed using quadrupole time-of-flight tandem mass spectrometer (Q-TOF Maxis, Bruker Daltonics, Germany) with an updated collision cell for electrospray ionization source.

| **Compound name (Negative mode)** | **m/z** | **Δppm** | **Adduct** |
| --- | --- | --- | --- |
| S-DGD-5-PA (1-O-[6-O-(2,3-di-O-phytanyl-1-phospho-sn-glycerol)-2-O-(2-HSO3-alpha-D-mannopyranosyl)-alpha-D-glucopyranosyl]-2,3-di-O-phytanyl-sn-glycerol) | 1770.3878 | 17 | [M-H]**^-^** |
| S-DGD-5-PA | 1792.393 | 29 | [M+Na-2H]**^-^** |
| 3-HSO3-Gal-alpha1-6-Man-beta1-2-Glc-alpha1-1-[sn-2,3-di-O-phytanylglycerol]-6-[phospho-sn-2,3-di-O-phytanylglycerol] | 2006.4487 | 0 | [M+CH3COO]**^-^** |
| Lipid IVA | 1946.4822 | 28 | [M-H]**^-^** |
|  | 1992.3818 | 24 | [M+FA-H]**^-^** |
|  | 1385.8155 | 14 | [M-H2O-H]**^-^** |
| 3-Deoxy-D-manno-octulosonyl-lipid IV(A) | 1623.8715 | 20 | [M-H]**^-^** |
| Lipid A -disaccharide-1-P | 1345.8289 | 24 | [M+Na-2H]**^-^** |
| GalNAcalpha1-3GalNAcbeta1-3Galalpha1-4Galbeta1-4Glcbeta-Cer(d18:1/24:0) | 1578.9478 | 28 | [M+K-2H]**^-^** |
| BPG(C20,C20,C20,C20) изомер (sn-2,3-di-O-phytanyl-1-phosphoglycerol-3-phospho-sn-2,3-di-O-phytanylglycerol) | 1520.2549 | 28 | [M-H]**^-^** |
| CL(1'-[14:1(9Z)/14:1(9Z)],3'-[14:1(9Z)/15:1(10Z)])[rac] | 1279.7775 | 18 | [M+Cl]**^-^** |
| BPG(C25,C20,C20,C20) | 1572.3935 | 17 | [M-H2O-H]**^-^** |
| KDNalpha2-3Galbeta1-4Glcbeta-Cer(d18:1/24:1(15Z)) | 1650.3505 | 28 | [M+CH3COO] |
|  | 1220.7617 | 22 | [M-H]**^-^** |
| Galbeta1-3GalNAcbeta1-3Galalpha1-4Galbeta1-4Glcbeta-Cer(d18:1/18:0) | 1280.7759 | 26 | [M+CH3COO]**^-^** |
|  | 1397.8087 | 4 | [M-H2O-H]**^-^** |
| Galalpha1-3Galalpha1-4Galbeta1-4Glcbeta-Cer(d18:1/22:0) | 1268.778 | 24 | [M-H]**^-^** |
| GalNAcbeta1-3Galalpha1-4Galbeta1-4Glcbeta-Cer(d18:1/20:0) | 1327.7937 | 12 | [M+FA-H]**^-^** |
| Fucalpha1-2Galalpha1-3Galalpha1-4Galbeta1-4Glcbeta-Cer(d18:1/20:0) | 1386.8113 | 18 | [M-H]**^-^** |
| GalNAcbeta1-4(NeuAcalpha2-3)Galbeta1-4Glcbeta-Cer(d18:1/24:0 | 1446.8242 | 23 | [M+CH3COO]**^-^** |
|  | 1504.8421 | 16 | [M+K-2H]**^-^** |
| Fucalpha1-2Galbeta1-3GalNAcbeta1-3Galalpha1-4Galbeta1-4Glcbeta-Cer(d18:1/16:0) | 1515.8397 | 1 | [M-H2O-H]**^-^** |
| Galbeta1-3Galalpha1-4Galbeta1-4Glcbeta-Cer(d18:1/20:0) | 1286.8121 | 21 | [M+FA-H]**^-^** |
| Galalpha1-3Galalpha1-3Galalpha1-4Galbeta1-4Glcbeta-Cer(d18:1/16:0) | 1328.7925 | 26 | [M-H2O-H]**^-^** |
| GalNAcalpha1-3GalNAcbeta1-3Galalpha1-4Galbeta1-4Glcbeta-Cer(d18:1/18:0) | 1368.7745 | 17 | [M+Na-2H]**^-^** |
|  | 1456.8245 | 19 | [M-H]**^-^** |
| alNAcbeta1-4(NeuGcalpha2-3)Galbeta1-4Glcbeta-Cer(d18:1/18:0) | 1398.8076 | 2 | [M-H]**^-^** |
| NeuAcalpha2-3GalNAcbeta1-3Galalpha1-4Galbeta1-4Glcbeta-Cer(d18:1/22:0) | 1622.8733 | 25 | [M+Na-2H]**^-^** |
| Galbeta1-4(NeuAcalpha2-3)Galbeta1-4Glcbeta-Cer(d18:1/18:0) | 1379.7754 | 21 | [M+K-2H]**^-^** |
| KDNalpha2-3Galbeta1-3GalNAcbeta1-4Galbeta1-4Glcbeta-Cer(d18:1/18:0) | 1485.8083 | 15 | [M-H2O-H]**^-^** |
| Fucalpha1-2Galalpha1-3Galalpha1-4Galbeta1-4Glcbeta-Cer(d18:1/18:0) | 1404.8437 | 23 | [M+FA-H]**^-^** |
| GalNAcbeta1-3Galalpha1-4Galbeta1-4Glcbeta-Cer(d18:1/20:0) | 1319.7614 | 0 | [M+K-2H]**^-^** |
| Galbeta1-3GalNAcbeta1-3Galalpha1-4Galbeta1-4Glcbeta-Cer(d18:1/16:0 | 1387.8084 | 9 | [M-H]**^-^** |
| M(IP)2C(t20:0/26:0(2OH)) | 1444.8281 | 12 | [M+CH3COO]**^-^** |
| NeuAcalpha2-3GalNAcbeta1-3Galalpha1-4Galbeta1-4Glcbeta-Cer(d18:1/18:0) | 1544.8239 | 29 | [M-H]**^-^** |
| GalNAcbeta1-3Galalpha1-4Galbeta1-4Glcbeta-Cer(d18:1/24:1(15Z)) | 1357.8236 | 7 | [M+Na-2H]**^-^** |
| DGDG(18:2(9Z,12Z)/18:2(9Z,12Z)[15(R)OH-18:2(9Z,12Z]) | 1239.793 | 14 | [M+Na-2H] |
| Fucalpha2-3GlcNAcbeta1-6GalNAcbeta1-3Galalpha1-4Galbeta1-4Glcbeta-Cer(d18:1/16:0) | 1574.8559 | 15 | [M-H]**^-^** |
| KDNalpha2-3Galbeta1-4(Fucalpha1-3)GlcNAcbeta1-3Galbeta1-4Glcbeta-Cer(d18:1/16:0) | 1603.8393 | 11 | [M-H2O-H]**^-^** |
| Galalpha1-3Galalpha1-4Galbeta1-4Glcbeta-Cer(d18:1/24:0) | 1334.8292 | 23 | [M+K-2H]**^-^** |
| Fucalpha1-2Galalpha1-3Galbeta1-4Glcbeta-Cer(d18:1/20:0) | 1334.8292 | 23 | [M+K-2H] |
|  | 1208.7637 | 20 | [M-H]**^-^** |
| NeuAcalpha2-3Galbeta1-3GalNAcbeta1-4(NeuAcalpha2-8NeuGcalpha2-3)Galbeta1-4Glcbeta-Cer(d18:1/26:0) | 1127.0957 | 8 | [M-2H]**^2-^** |
| Galalpha1-3Galalpha1-3Galalpha1-4Galbeta1-4Glcbeta-Cer(d18:1/18:0) | 1374.8114 | 8 | [M-H]**^-^** |
| Fucalpha1-2Galalpha1-3Galalpha1-4Galbeta1-4Glcbeta-Cer(d18:1/24:0 | 1464.8589 | 15 | [M+Na-2H]**^-^** |

**Supplementary Table 3** The binding energies of the ligands (PC- phosphatidylcholine, PE- phosphatidylethanolamine, PSC - Polysaccharide chain, Lipid A ) with pBFT2 and mBFT2, resulting docking via ICM

| **mBFT2** | **Ligands** | **∆*Gbind***  ***(kcal /mol)*** | **∆*Gel***  ***(kcal /mol)*** | **∆*Gpolar (kcal /mol)*** | **∆*Gnonpolar (kcal /mol)*** | ***T*∆*S***  ***(kcal /mol)*** |
| --- | --- | --- | --- | --- | --- | --- |
|  | PE | -11,21 | -17,51 | 15,17 | -13,37 | -4,50 |
|  | PC | -5,86 | -11,45 | 14,85 | -14,46 | -5,20 |
|  | Lipid A | -10,68 | -9,80 | 15,20 | -24,30 | -8,22 |
|  | PSC with PO2 | -8,94 | -14,04 | 15,76 | -16,68 | -6,02 |
|  | PSC without PO2 | -11,31 | -9,97 | 9,60 | -17,54 | -6,60 |
| **pBFT2** | PE | -7,78 | -5,44 | 8,21 | -16,97 | -6,42 |
|  | PC | -6,90 | 3,15 | 2,91 | -19,78 | -6,82 |

**SUPPLEMENTARY TEXT**

**SDS PAGE**

Cells fractions from ETBF and NTBF were mixed with Laemmli sample buffer (1:1) containing CHAPS and separated by SDS-PAGE. 40 µg of each fraction were boiled for 10 min prior to electrophoresis. Gels were stained with Coomassie brilliant blue stain

**In-gel trypsin digestion of protein samples**

Gel were cut into small (1 × 1 mm) pieces and transferred into sample tubes. Protein disulfide bonds were reduced with 10 mM DTT (in 100 mM ammonium bicarbonate buffer) at 50^○^ C for 30 min and afterwards alkylated with 55 mM iodoacetamide (in 100 mM ammonium bicarbonate buffer) at room temperature for 20 min in the dark. After alkylation, gel samples were stained with 50% ACN (in 50 mM ammonium bicarbonate buffer) and dehydrated by addition of 100% ACN. After removal 100% ACN, the samples were subjected to the in-gel trypsin digestion. The digestion buffer contained 13 ng/µl trypsin (in 50 mM ammonium bicarbonate buffer). The trypsin digestion proceeded overnight at 37^○^ C. The resulting tryptic peptides were extracted from the gel by adding two volumes of 0.5% TFA into the samples (incubation during1 h), and, then, two volumes of 50% ACN (incubation during 1 h). Finally, the extracted peptides were dried in vacuum and redissolved in 3% ACN with 0.1% FA solution prior to LC-MS/MS analysis.

**Search Database Creation**

ProtDB search database was created using the annotated proteins of *Bacteroides fragilis* BOB25. Annotated proteins were downloaded in fasta format (RefSeq: NZ_CP011073.1, 4127 amino acid sequences). The genome was downloaded from NCBI in fasta format (NZ_CP011073.1) and was translated in 6-frames. Stop-to-stop ORFs were exported using the Artemis software version 16.0.0 with option “Mark Open Reading Frames”. Minimal ORF length was set at 40 amino acids.

**Proteins and Peptides Identification**

Raw data files with WIFF and .D file format were converted to the Mascot generic format (MGF file format) using AB SCIEX MS Data Converter version 1.3 and Compass Data Analysis 4.2 (Build 383.1) respectively. The proteins identification was carried out using Mascot Search Engine version 2.5.1.The Mascot searches were performed with the following parameters: tryptic-specific peptides, maximum of one missed cleavages, a peptide charge state limited to 1+,2+ and 3+, a peptide mass tolerance of 10 ppm, a fragment mass tolerance of 0.5 Da, variable modifications caused by Oxidation(M) and Propionamide(C). The decoy search strategy to calculate FDR was used. The score threshold was calculated using Mascot. Individual ions score higher than score threshold indicate identity or extensive homology with p < 0.05 and FDR < 5%. A peptide was identified if its rank was 1 and score higher than score threshold. A protein was identified if has 2 and more identified unique peptides.

**Modeling and docking procedure**

The amino acids substitutions in the pBFT3 model were made to yield a pBFT2 model: Asn102 → Ser102, Tyr169 → Asp169, Pro170 → Leu170, Val177 → Leu177, Asn228 → Ser228, Tyr257 → Phe257, Ala270 → Ser270, Asn320 → Asp320, Asn357 → Arg357 and Asp383 → Tyr383. The processed form of the toxin (mBFT2) was obtained by removing amino acid residues from 221 to the C-terminus. The 3D models of the targets (PE, PC, Lipid A, and polysaccharide chain of LPS) were created with Molsoft ICM version 3.8-3 application (43, 44). Docking was carried out in two stages. In the first step search for the most likely binding sites was carried out on the rigid targets. The target-ligand complexes obtained from the first rigid-body docking were further refined by optimizing of the conformation of side-chains amino acids located in 4 Å radius vicinity of the ligand with application BPMC procedure. The binding energy scoring used in these stages and BPMC procedure are described by Totrov M. (32). Finally confomational stack obtained from refining docking procedure was sorted by energy, and 100 best conformations were taken for more accurately energy of binding estimate. The binding energy was estimated as the difference between of the free energy of the dissolved complex value and the sum of the free energies of dissolved unbound target and ligand values. When evaluating the Van der Waals contributions and hydrogen bonds to the binding energy the same assumptions which discussed in the article (48) were used as a basis. As a result of the foregoing, binding energy is calculated using the formula:

∆G_bind_=∆G_el_+∆G_polar_ +∆G_nonpolar_- T∆S

Unlike to the docking procedure, during which the value of solvation energy is calculated using the method proposed by Wesson and Eisenberg (49), for the conformation stored in the stack, it is calculated more accurately - as the sum of the contribution ∆G_polar_ polar term (resulting from desolvation of partial charges transferred from aqueous medium to the receptor-binding cavity) and non polar term ∆G_nonpolar_ (including a solute–solvent the Van der Waals interaction and the value of energy required for forming cavity being equal of solute volume in solvent). Since estimation of electrostatic energy in docking procedure was performed quite simplistically, so coulomb interactions and ∆G_polar_ were calculated by solving the Poisson equation with a rapid-exact-boundary element (REBEL) method (50). According to recommendations of ICM developers, the dielectric constants of the targets, the ligands and the complexes were taken as 12.7; the respective parameter for implicit water was 78.5. The hydrophobic component ∆G_nonpolar_ was calculated as a product of the total solvent accessible area by the surface tension parameter (0,012 kcal/(mol*Å^2^). The entropic term - T∆S was estimated as sum of conformational entropy of the ligand and target. The ligand conformational entropy is calculated as RTLn(Ns), where Ns is the number of low-energy conformational states generated by Monte Carlo simulations. The loss of configurational entropy of the targets upon binding to the ligands was calculated using maximal possible entropy read from the software residue library for side chains of amino acid involved in binding with ligands.

**SUPPLEMENTARY FIGURES**

**
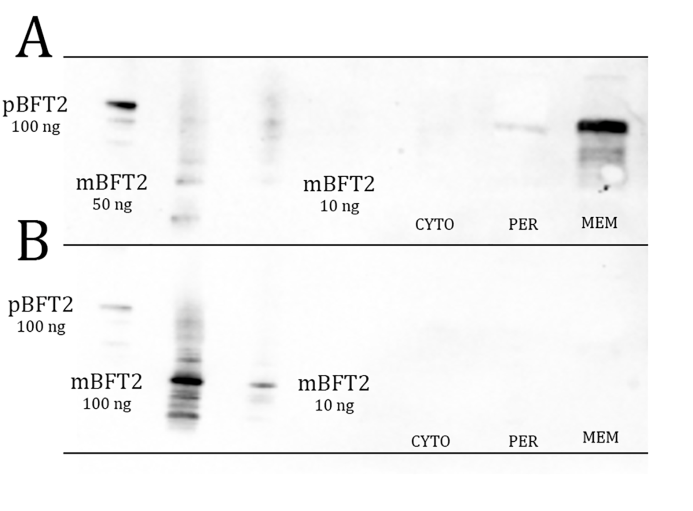
**

**Supplementary Fig. 1 Analyses of BFT localization in subcellular B. fragilis fractions.** 1 µg of each ETBF **(A)** and NTBF **(B)** cell fractions (CYTO - cytoplasmic fraction, PER -periplasmic fraction and MEM - membrane fraction respectively) were run on 10% SDS-PAGE followed by Western blotting with antibody against BFT2.

**
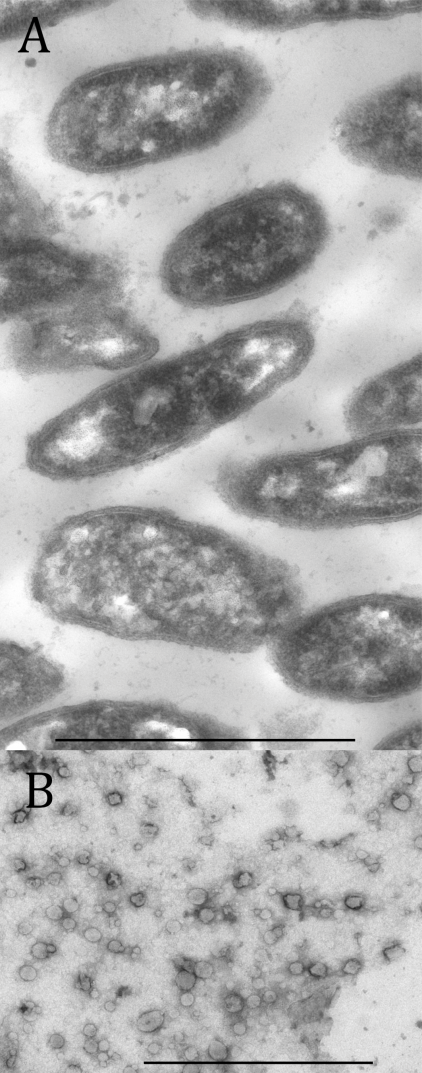
**

**Supplementary Fig. 2** - TEM of thin section of NTBF cells and negatively stained OMV preparations isolated from NTBF culture contain no fixed labels coupled with antibodies against BFT. Scale bars represent 1 µm for **(A)** and 600 nm for **(B)**.

**
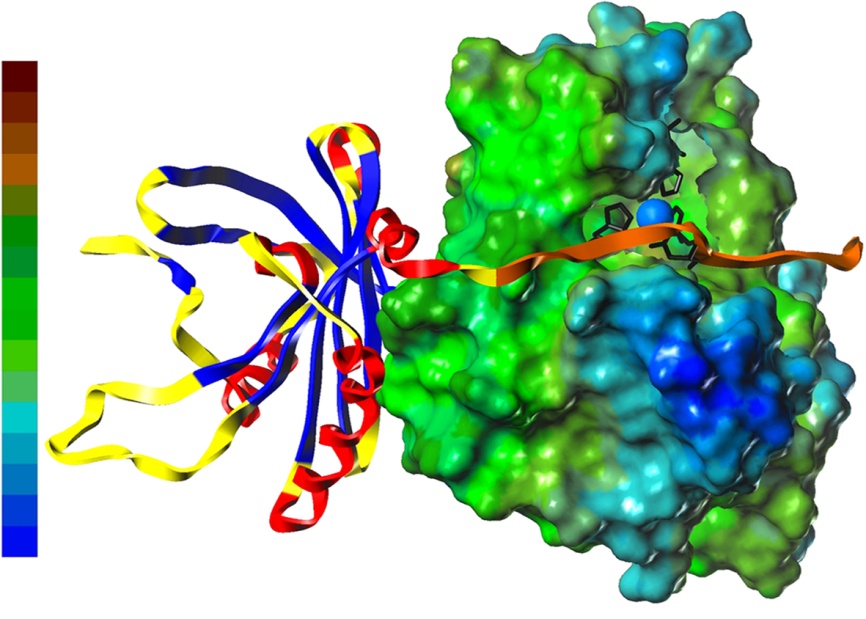
**

**Supplementary Fig. 3 Modeling of mBFT2 surface according hydrophobic potential distribution.** Conolly surface of mBFT2 colored according hydrophobic potential distribution and fragment of pBFT2 (amino acid residues Pro187 - Val201- β13 is highlighted in terracotta) highlighted rendering. β13 is located in proximity to the active center of the catalytic domain and prevents any interactions with the active center due to steric hindrance (created by SYBYL X1.2.). Carbonyl oxygen atoms of the β13 fragment form coordination bonds with the zinc ion in the active center. The scale to the left shows the distribution of hydrophobic potential from negative (blue) to positive (dark red)


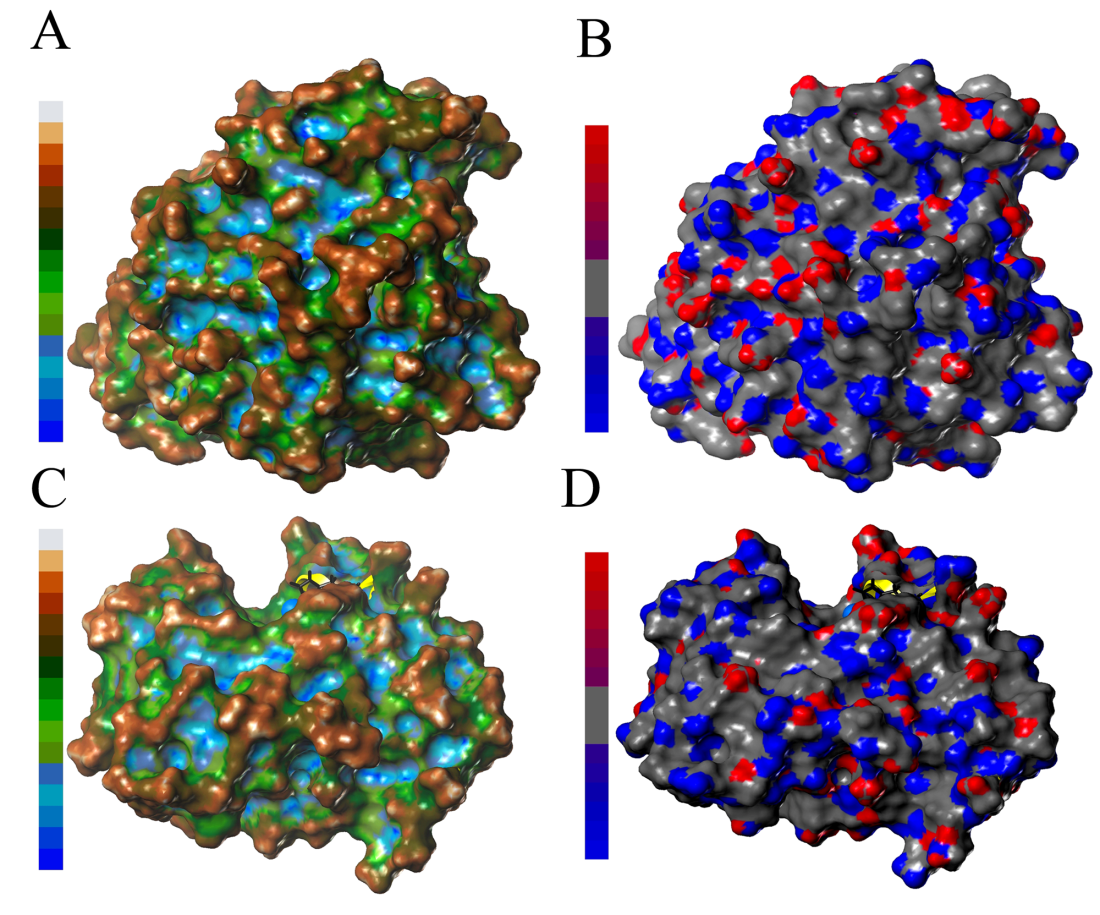


**Supplementary Fig. 4 Modeling of pBFT2 and mBFT2 surface according cavities (A,C) and potential donors and acceptors of hydrogen bonds distribution (B,D). (A,C) -** Conolly surface of pBFT2 and mBFT2 colored according cavities distribution (created by SYBYL X1.2); Distribution of cavities depended of deep, from the surface (grey) to deeper (blue). **(B-D)** pBFT2 and mBFT2 colored according potential donors and acceptors of hydrogen bonds distribution on the protein surface (created by SYBYL X1.2.). The scale to the left shows the distribution of potential donors and acceptors of hydrogen bonds, acceptors – blue, donors – red.


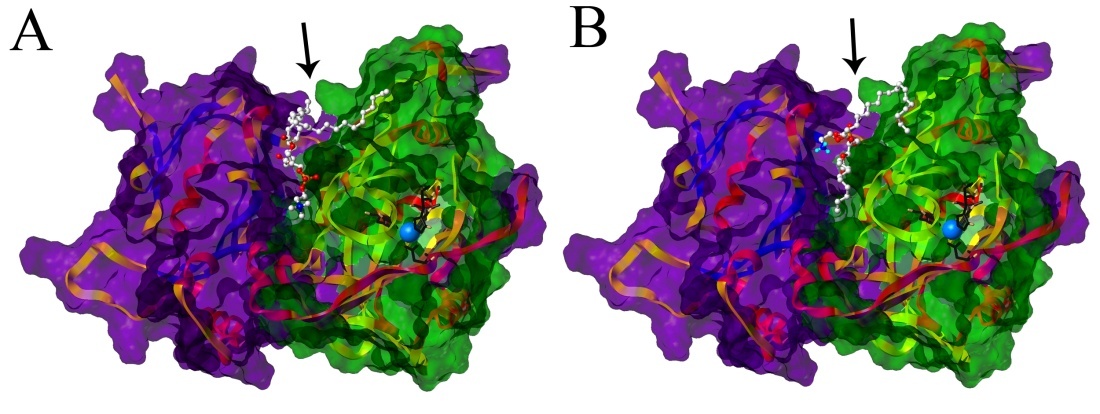


**Supplementary Fig. 5** **Modeling and docking of pBFT2-lipid association.** Structure model of pBFT2 and best (according to the binding energy) PC **(A)** and PE **(B)** location on the pBFT2 Conolly surface, resulting in docking via ICM. Connolly surface painted in lilac for the part removed during processing and in green for the part that carries the active site of the protein. Rendering of mBFT2 is colored in accordance with the secondary structure, Zn ions are painted in blue and amino acids (Asp194, His348, His352, His358), associated with ion by coordination bonds in black. The atoms of the ligands are painted in the following colors: polar hydrogens - blue, nitrogen - dark blue, oxygen - red phosphorus - orange, carbon - white. The non-polar hydrogens not shown.


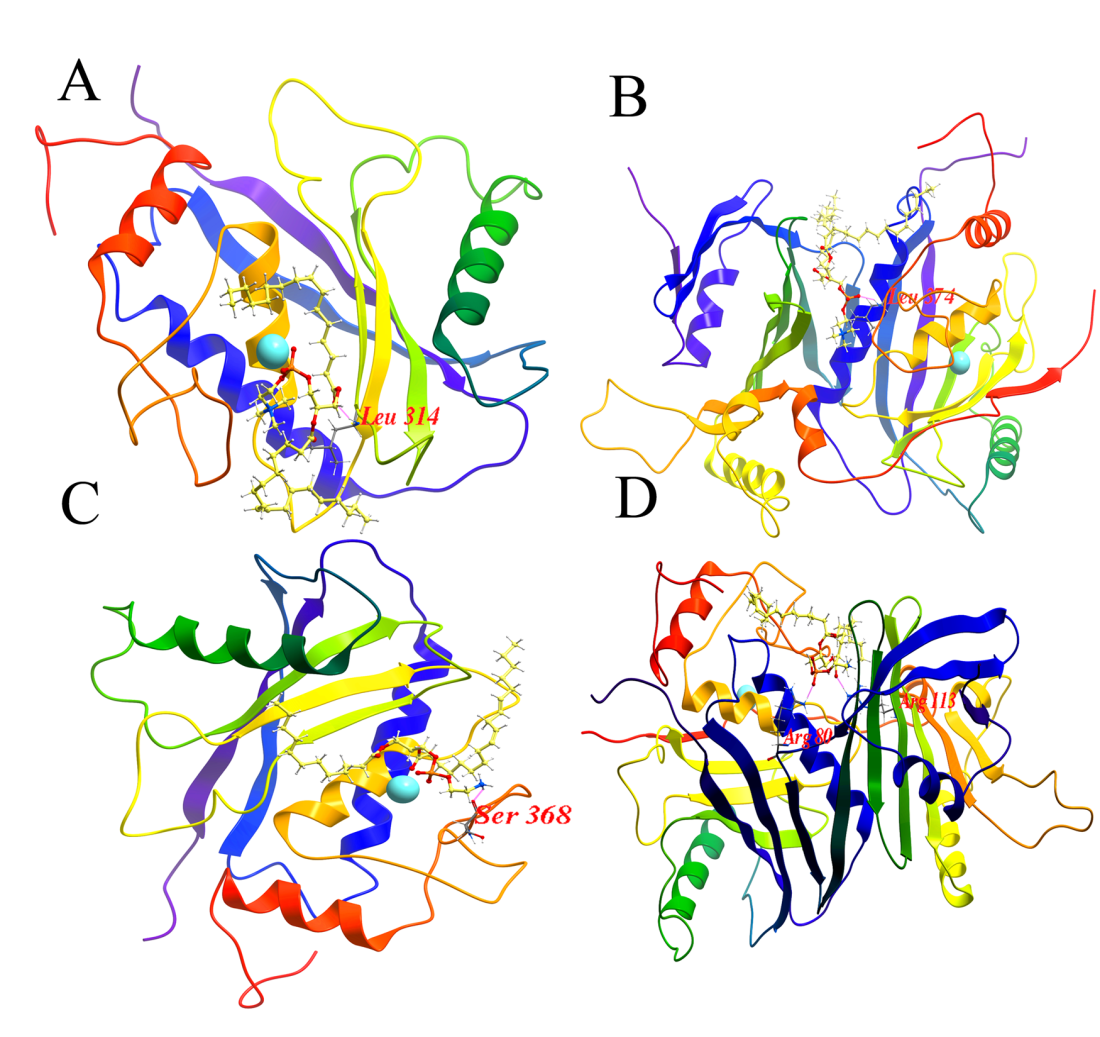


**Supplementary** **Fig. 6 Detailed image of mBFT2 active center and PC (A,B) or PE (C,D) interaction**. Zinc ion painted blue. Oxygen of lipid phosphate group colored in red forming coordination bonds with zinc ion. Hydrogen bonds, formed between single amino acids of the toxin and lipids phosphate group oxygen are colored in pink.
